# Enhanced CO_2_ Methanation over Solution-Combustion Synthesized Ni/Kaolin Catalysts: The Effect of Fe and La Promotion

**DOI:** 10.3390/molecules31183218

**Published:** 2026-09-11

**Authors:** Agnieszka Szymaszek-Wawryca, Szymon Hanf, Michał Szymaszek, Konrad Świerczek, Dorota Duraczyńska, Mateusz Marzec, Monika Motak

**Affiliations:** 1 Faculty of Energy and Fuels, AGH University of Krakow, Al. A. Mickiewicza 30, 30-059 Kraków, Poland; szhanf@student.agh.edu.pl (S.H.); szymaszek@student.agh.edu.pl (M.S.); xi@agh.edu.pl (K.Ś.); motakm@agh.edu.pl (M.M.); 2 Jerzy Haber Institute of Catalysis and Surface Chemistry, Polish Academy of Sciences, Niezapominajek 8, 30-239 Kraków, Poland; dorota.duraczynska@ikifp.edu.pl; 3 Academic Centre for Materials and Nanotechnology, AGH University of Krakow, Ul. Kawiory 30, 30-055 Kraków, Poland; marzecm@agh.edu.pl

**Keywords:** CO_2_ methanation, nickel catalysts, iron catalysts, lanthanide promoters, solution combustion synthesis, kaolin

## Abstract

Ni-based catalysts supported on kaolin were synthesized via solution combustion synthesis and promoted with Fe and/or La to investigate their catalytic performance in CO_2_ methanation. Catalytic tests showed that Fe significantly improved CO_2_ conversion from approximately 52% for Ni-catalyst to 83% for Ni2.5Fe_SCS at 300 °C. However, the promotional effect of Fe showed only a weak dependence on its loading, with Ni modification already being achieved at the lowest Fe content. Among all investigated catalysts, the La-promoted sample exhibited the highest CO_2_ conversion (85% at 300 °C), owing to the formation of highly dispersed Ni^0^ crystallites, enhanced surface basicity, and strong metal–support interactions. These interactions also effectively suppressed particle sintering during prolonged stability tests. In contrast, simultaneous promotion with Fe and La did not provide additional catalytic enhancement, indicating that the effects of both promoters were not simply additive.

## 1. Introduction

Carbon dioxide (CO_2_) emissions represent one of the major environmental challenges associated with the ongoing demand for energy and industrial development [1]. Nevertheless, fossil fuels remain the principal source of CO_2_ emissions and their consumption is expected to increase by approximately 2% by 2030. In response, international initiatives, such as the Kyoto Protocol, the Paris Agreement, and the EU’s climate strategies have been established to promote the transition toward climate neutrality [2,3]. One promising approach is to combine carbon capture and utilization (CCU) with power-to-gas (PtG) [4]. This strategy involves producing hydrogen (H_2_) via renewable-powered electrolysis and capturing CO_2_ from industrial sources, followed by their conversion to methane (CH_4_) through the Sabatier reaction (Equation (1)) [5]. Unlike hydrogen, methane can be directly injected into existing natural gas grids, offering greater versatility.

(1) $${CO}_{2}+4H_{2}⇌{CH}_{4}+2H_{2}O;\;\Delta {H{}^{\circ}}_{298K}=-165\;kJ/mol$$

The Sabatier reaction, or catalytic methanation of CO_2,_ is an exothermic process typically conducted at 250–500 °C and is accompanied by a significant volume contraction of approximately 40% [6]. In spite of its thermodynamic favorability (Δ${G{}^{\circ}}_{298K}$ = −131 kJ/mol), CO_2_ methanation requires elevated pressures and moderate temperatures, posing significant economic and technical challenges. Moreover, the linear structure and fully oxidized state of CO_2_ make its hydrogenation to CH_4_ kinetically demanding due to the high energy required for CO_2_ activation [7,8]. At low temperatures and high pressures, the competitive reverse water-gas shift (RWGS) reaction may also occur, leading to CO formation (Equation (2)) [9]:

(2) $${CO}_{2}+H_{2}⇌CO+H_{2}O;\;\Delta {H{}^{\circ}}_{298K}=+41\;kJ/mol$$

Moreover, the Boudouard reaction (Equation (3)), which leads to the generation of carbon deposits, can gradually deactivate the catalyst [10].

(3) $${2CO⇌CO}_{2}+C_{\left(solid\right)};\;\Delta {H{}^{\circ}}_{298K}=-41\;kJ/mol$$

Due to these challenges, CO_2_ methanation requires highly active, selective, and stable catalysts with optimized chemical composition and structure. Considering the Sabatier reaction, metal-based supported catalysts with noble metals show superior performance. Nevertheless, their high cost and short supply limit large-scale industrial use [11]. The cost-effective alternatives, which are extensively investigated, are nickel (Ni)-based catalysts [12,13,14,15]. Nonetheless, their important limitations have driven the development of multimetallic systems.

According to the literature, iron (Fe)-based catalysts exhibit good catalytic performance, low toxicity, and relatively low cost [16,17,18]. However, monometallic Fe-catalysts generally exhibit low CH_4_ selectivity, which limits their commercial application. For example, Kirchner et al. [17] reported that the CO_2_ methanation performance of unsupported iron oxides (α-Fe_2_O_3_, β-Fe_2_O_3_, and γ-Fe_2_O_3_) strongly depends on the oxide phase and the nature of carbon species formed during the reaction. While reactive surface carbon enhanced the activity of γ-Fe_2_O_3_, less reactive carbon species formed on α- and β-Fe_2_O_3_ lowered CH_4_ yields. Furthermore, single Fe-based catalysts are widely used in Fischer-Tropsch synthesis, favoring the formation of hydrocarbons higher than methane [19,20].

To overcome the above-mentioned drawbacks, iron is often combined with nickel, leading to more efficient methanation catalysts. A number of studies have shown that the supported Ni-Fe systems exhibit improved catalytic performance due to the increased dispersion of nickel and better stability compared to single Ni-catalysts [21,22,23]. It was also reported that the synergistic effect of Ni-Fe facilitates adsorption of carbon dioxide due to the increased surface basicity provided by iron [24]. Based on the density functional theory (DFT) simulations conducted by Andersson et al. [25], Ni-Fe alloys contribute to the optimal C-O dissociation energies, leading to the excellent methanation activity. Hwang et al. [26] investigated Ni-Fe-Al_2_O_3_ xerogel catalysts and assigned their satisfactory performance to improved reducibility in the presence of Fe.

To further enhance CO_2_ methanation activity, Ni-based catalysts can be promoted with rare-earth metals, such as lanthanum (La) [27,28,29]. In general, the introduction of La increases the concentration of the medium basic sites and promotes the CO_2_ associative pathway [30]. Moreover, La was reported to reduce Ni^0^ crystallite size, facilitating selectivity to methane [27,28]. Wierzbicki et al. [27] investigated La-promoted hydrotalcite-derived Ni-catalysts for CO_2_ methanation using operando XANES and XES spectroscopy. The authors showed that La modifies the Ni–oxide matrix interaction and electronic structure, while improving Ni reducibility, CO_2_ adsorption, and surface basicity. Zhang et al. [28] confirmed that 5 wt.% La was optimal for improving Ni dispersion and low-temperature CO_2_ methanation activity over Ni/Mg-Al catalysts. Hoang Ho et al. [29] confirmed that Fe and La promote Ni-catalysts through different effects: Fe modifies the Ni active phase, whereas La helps maintain small Ni^0^ particles and enhances surface basicity. While Fe- and La-promoted Ni catalysts have been extensively studied individually, their combined effects in CO_2_ methanation remain poorly understood. In particular, the structure–performance relationships in Ni–Fe–La systems have not been sufficiently explored, despite the potential of combining the distinct promotional effects of Fe and La. Therefore, systematic investigation of the combined influence of Fe and La on Ni-based catalysts is required to evaluate their individual and cooperative effects.

One of the key factors determining the performance of CO_2_ methanation catalysts is the dispersion of the active species, which can be challenging to maintain in multimetallic systems. Solution combustion synthesis (SCS) is a promising single-step approach for the preparation of mixed-metal oxides, as it enables high metal loadings while maintaining good dispersion and structural integrity [11,31,32]. Typically, SCS involves the combustion of a solution containing metal precursors, usually nitrates, and a fuel such as urea, glycine, or citric acid, performed at 300–600 °C. The process is based on a redox reaction governed by complex stoichiometric relationships [33]. Depending on the composition of the reaction mixture and the energy supplied to initiate combustion, different products can be obtained, including metal nanooxides and mixtures of gases [34]. Despite many advantages, the application of SCS for the preparation of supported Ni-Fe oxide catalysts promoted with La has, to the best of our knowledge, received very limited attention.

**Figure 1:**
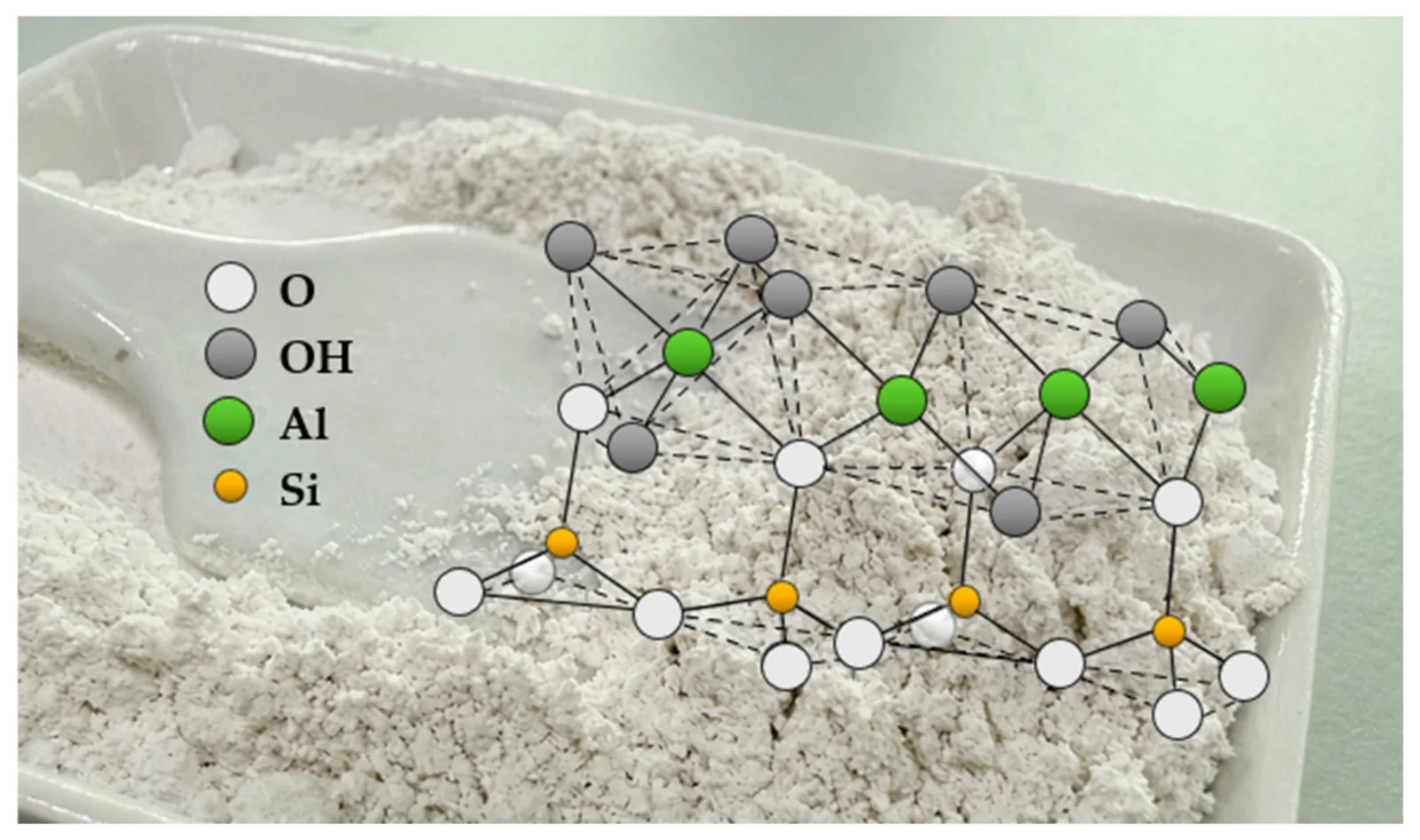
Schematic representation of the kaolinite structure.

The performance of supported catalysts is also strongly influenced by the nature of the support. In addition to conventional supports, such as SiO_2_, Al_2_O_3_, and TiO_2_ [35,36,37], naturally occurring aluminosilicates offer an attractive alternative due to their low cost, environmental benefits, and availability. Natural kaolin, consisting predominantly of kaolinite, with smaller amounts of other minerals, such as smectite, mica, and quartz, exhibits a highly crystalline, hydrated, layered aluminosilicate structure [38,39]. The clay is particularly promising as a catalyst support because of its high structural stability and the presence of alkali and alkaline-earth metal oxides. Despite these favorable properties, the potential of kaolin as a support for CO_2_ methanation catalysts remains largely unexplored and underutilized. The schematic structure of kaolinite is presented in Figure 1.

The high crystallinity and chemical inertness of natural kaolin generally necessitate a preliminary activation step, most commonly achieved through thermal treatment. Typically, the thermal transformation of kaolin proceeds through the dehydroxylation of kaolinite at approximately 600–900 °C, resulting in the formation of a predominantly amorphous metakaolin phase. Upon further heating, structural reorganization of the oxide network occurs, with the formation of crystalline γ-Al_2_O_3_ at around 1100 °C, followed by the crystallization of mullite and cristobalite at approximately 1400 °C [40]. Ayodele et al. [39] applied acid or a base treatment of natural kaolin as a support for the dry reforming of methane (DRM). Wang et al. [8] used thermally treated kaolin to prepare Ni-Ce catalysts for hydrothermal gasification of biomass to produce hydrogen, while Boukhemkhem et al. [41] investigated non-modified kaolin as a support for Fe catalysts in wet peroxide oxidation. These studies demonstrate the potential of kaolin as a catalyst support. However, its application in CO_2_ methanation remains limited.

Overall, while the individual promotional effects of Fe and La in Ni-based catalysts for CO_2_ methanation have been extensively studied, their combined influence remains poorly understood. In particular, the interplay between Fe and La and its effect on the structure, surface properties, and catalytic performance of Ni-based catalysts have received limited attention. Therefore, this study systematically compares Ni, Ni–Fe, Ni–La, and Ni–Fe–La catalysts supported on thermally treated kaolin, providing insights into the distinct and combined roles of Fe and La in CO_2_ methanation. The work highlights the unexplored potential of combining SCS with underexplored natural kaolin support for the development of efficient, suitable, and economically attractive catalysts for CO_2_ valorization.

## 2. Results

### 2.1. Physicochemical Characterization of the Catalysts

#### 2.1.1. Chemical Composition, Crystalline Structure, and Morphology

The chemical composition of the catalysts was determined using point energy-dispersive X-ray spectroscopy (EDS) coupled with scanning electron microscopy (SEM), performed at selected locations on the sample surface. The obtained results are collected in Table 1. EDS analysis confirmed the presence of Si, Al, and Mg originating from the aluminosilicate support, as well as the introduced Ni in all investigated samples. For Fe-modified catalysts, the iron content increased from 2.5 wt.% for Ni2.5Fe SCS to 5 wt.% for Ni5Fe SCS and 7 wt.% for Ni7.5Fe SCS, confirming the successful incorporation of iron into the catalyst composition. The La-promoted samples additionally exhibited La signals, with the La content remaining in the range of approximately 3–4 wt.%. Due to the local and semi-quantitative character of the EDS technique, slight deviations from nominal compositions may be related to the heterogeneous morphology and point-specific nature of the analysis.

**Figure 2:**
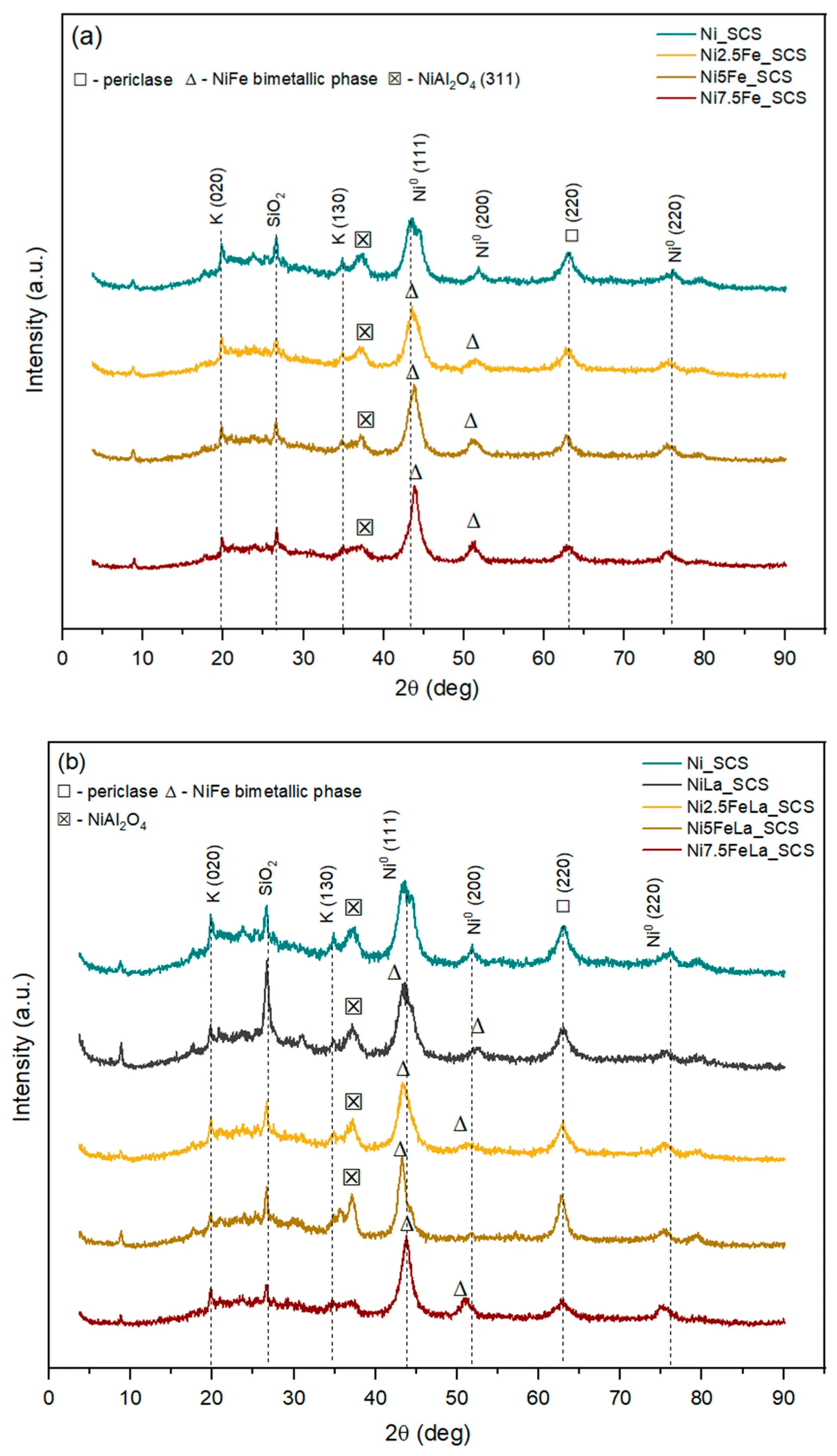
XRD patterns obtained for the reduced samples: kaolin modified with Ni and Fe (**a**); kaolin modified with Ni, Fe, and La (**b**).

The crystalline structure of non-modified kaolin and the catalysts was evaluated using X-ray diffraction (XRD). The XRD patterns obtained for the support, the calcined, as well as the reduced samples are presented in Figure S1, Figure S2 and Figure 2, respectively. The natural kaolin displays characteristic diffraction peaks assigned to kaolinite phase at 2*θ* = 12.32 (001), 19.72 (020), 20.85 ($\bar{1}$10), 25.06 (002), 35.04 (130), 38.45 ($\bar{1}$31), and 55.21° (300) [42]. The intense diffraction signal at 2*θ* = 26.6° corresponds to highly crystalline SiO_2_, in accordance with ICDD 46-1045. Typically, naturally occurring kaolin is composed of hydrate aluminosilicate. At high temperature, the clay transforms into metakaolin via dehydroxylation, which is presented in Equation (4).

(4) $${Si}_{2}{Al}_{2}O_{5}{\left(OH\right)}_{4}⟶{Al}_{2}O_{3}\cdot 2{SiO}_{2}+2H_{2}O$$

In general, dehydroxylation results in the removal of hydroxyl groups and release of water molecules, followed by a structural transformation or structural collapse [43]. Thus, the diffraction peaks characteristic of metakaolin appear in the XRD patterns of the calcined, spent, and reduced samples as broad bands within 2*θ* = 20–30°. As confirmed by the literature [44,45], the metakaolin phase exhibits higher reactivity, which might be beneficial for depositing nickel and iron onto its surface.

Figure S2 shows XRD patterns obtained for kaolin modified with Ni and Fe (a) and Ni, Fe, and La (b) after calcination. Regardless of the presence of iron or promotion with lanthanum, all samples exhibited distinct characteristic diffraction peaks of NiO (ICDD 47-1049) at 2*θ* = 43.3°. The result indicates that nickel in the calcined materials exists predominantly in the form of Ni^2+^, which is attributed to the combustion process followed by the calcination of Ni(NO_3_)_2_ to NiO. The outcomes are in line with the literature reports [46]. Moreover, no significant diffraction signals assigned to iron or lanthanum species were detected in the diffractograms, possibly due to their homogeneous dispersion, as confirmed by EDS mapping. In addition, regardless of the chemical state of the samples (calcined, reduced), there is a diffraction signal assigned to periclase-like structure at 62.3° (MgO, ICDD 03-065-2901) [11].

To gain deeper insight into the crystalline phases formed after reduction, the catalysts reduced in a gas mixture identical to that used during the catalytic tests were analyzed using ex situ XRD (Figure 2). The results indicated that no diffraction peaks assigned to crystalline NiO were observed, which confirmed the effective reduction of nickel oxide to metallic Ni^0^ under the applied conditions, regardless of Fe loading. The Ni_SCS sample exhibited characteristic sharp peaks centered at 2*θ* = 44.6° (111), 51.8° (200), and 76.4° (220), corresponding to metallic nickel (ICDD 03-065-0380). For the reduced NiFe catalysts, the diffraction peaks assigned to metallic Ni were slightly shifted toward lower 2*θ* values, regardless of the iron loading. This behavior suggests the formation of a Ni-Fe solid solution, which modified the lattice parameter of metallic nickel [46,47]. One can notice that no diffraction peaks corresponding to crystalline iron oxides were detected after reduction. This evidences that iron was either highly dispersed or fully reduced and incorporated into the Ni lattice. Additionally, the diffraction peak observed at 2*θ* = 37.6° after reduction is attributed to the (311) reflection of NiAl_2_O_4_ [47]. The spinel phase, most likely formed during calcination, became more evident after reduction owing to the disappearance of NiO reflections and changes in the relative intensities of the crystalline phases. Additionally, the intensity of the NiAl_2_O_4_ reflection remained nearly unchanged with increasing Fe content, suggesting that Fe incorporation did not significantly affect spinel formation. The diffraction peak at approximately 62.5°, corresponding to periclase (MgO), was preserved after reduction. This observation suggests that the Mg-containing oxide matrix remained structurally stable throughout the reduction process. As shown in Figure 2b, the introduction of La resulted in some noticeable changes in the XRD patterns. It is apparent that the diffraction peaks assigned to the Ni-Fe metallic phase for samples promoted with lanthanum are significantly less intense, particularly for 2.5 and 5 wt.% of Fe. This observation suggests that the addition of La improves the dispersion of Ni-Fe phase and/or suppresses the growth of large Ni-Fe crystallites at Fe loadings below 7.5 wt.%. The broader diffraction peaks observed for the La-promoted catalysts indicate the formation of smaller metallic crystallites, confirming that La effectively inhibits particle growth during reduction. Additionally, the results revealed that the diffraction signal related to NiAl_2_O_4_ became less intense for Ni7.5FeLa_SCS. The lower intensity of this peak suggests that La addition may inhibit the formation of large crystalline domains of NiAl_2_O_4_ or reduce their crystallinity.

To further evaluate the effect of Fe and La on the metallic phase, the crystallite size of Ni^0^ was calculated from the diffraction peaks using the Scherrer equation (Table 2). The NiO crystallite size ranged from 6.0 to 8.0 nm for most catalysts, with a value of 6.5 nm for the unpromoted Ni_SCS sample. For the Ni-Fe series, the crystallite size depended on the Fe loading, reaching a maximum of 8.0 nm for Ni5Fe_SCS, whereas the catalyst containing 7.5 wt.% Fe exhibited a crystallite size comparable to that of the monometallic Ni catalyst. The most pronounced reduction in NiO crystallite size was observed for the La-promoted NiLa_SCS catalyst (3.7 nm), indicating that La effectively inhibited NiO crystallite growth. A similar, although less pronounced, effect was also observed for the La-promoted Ni-Fe catalysts. The obtained results are in line with the literature: Hoang Ho et al. [29] prepared layered double hydroxides containing Ni, La, and Al via a co-precipitation method. Notably, the presence of lanthanum consistently led to a smaller crystallite size, regardless of the synthesis route, as a similar refining effect was observed in the present study where La was introduced via dry impregnation. The observed reduction in NiO crystallite size upon lanthanum addition can be attributed to the structural barrier effect and enhanced surface dispersion. Due to its larger ionic radius and high thermal stability, the lanthanum species (i.e., carbonates) act as physical spacers on the support. This separate phase effectively anchors the nickel oxide species and inhibits their surface migration and agglomeration during the calcination process. Furthermore, as evidenced by XRD patterns for the reduced samples (Figure 2b), activation with H_2_ produced Ni^0^ crystallites of 3.7 nm for Ni_SCS. For Ni-Fe catalysts the crystallite size increased linearly with Fe loading, while no change was observed after impregnation with La (NiLa_SCS). Interestingly, co-existence of iron and lanthanum resulted in increased Ni^0^ size and the effect was more pronounced at higher Fe contents. The particle growth facilitated by the formation of Ni-Fe phase combined with the possible strong interaction of Fe and La lowers the availability of the surface lanthanum species acting as spacers inhibiting nickel sintering. This particle growth can be directly correlated with the higher reduction temperatures of the La-promoted samples (see H_2_-TPR results). The increased thermal input during reduction accelerated surface diffusion and favored the growth of larger metal crystallites. As a consequence, lanthanum lost its ability to prevent particle growth, which together with Ni-Fe formation results in larger metallic domains.

**Figure 3:**
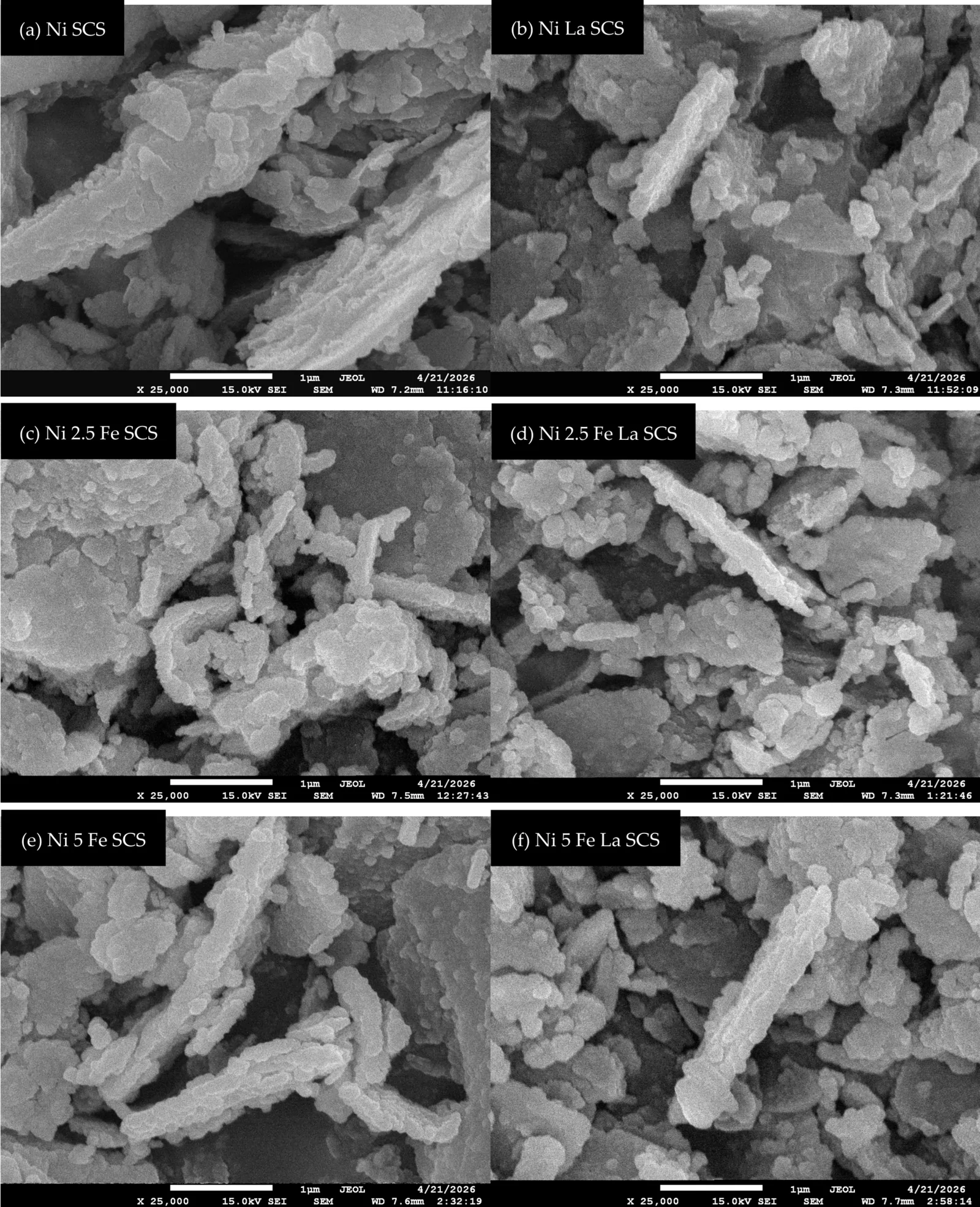

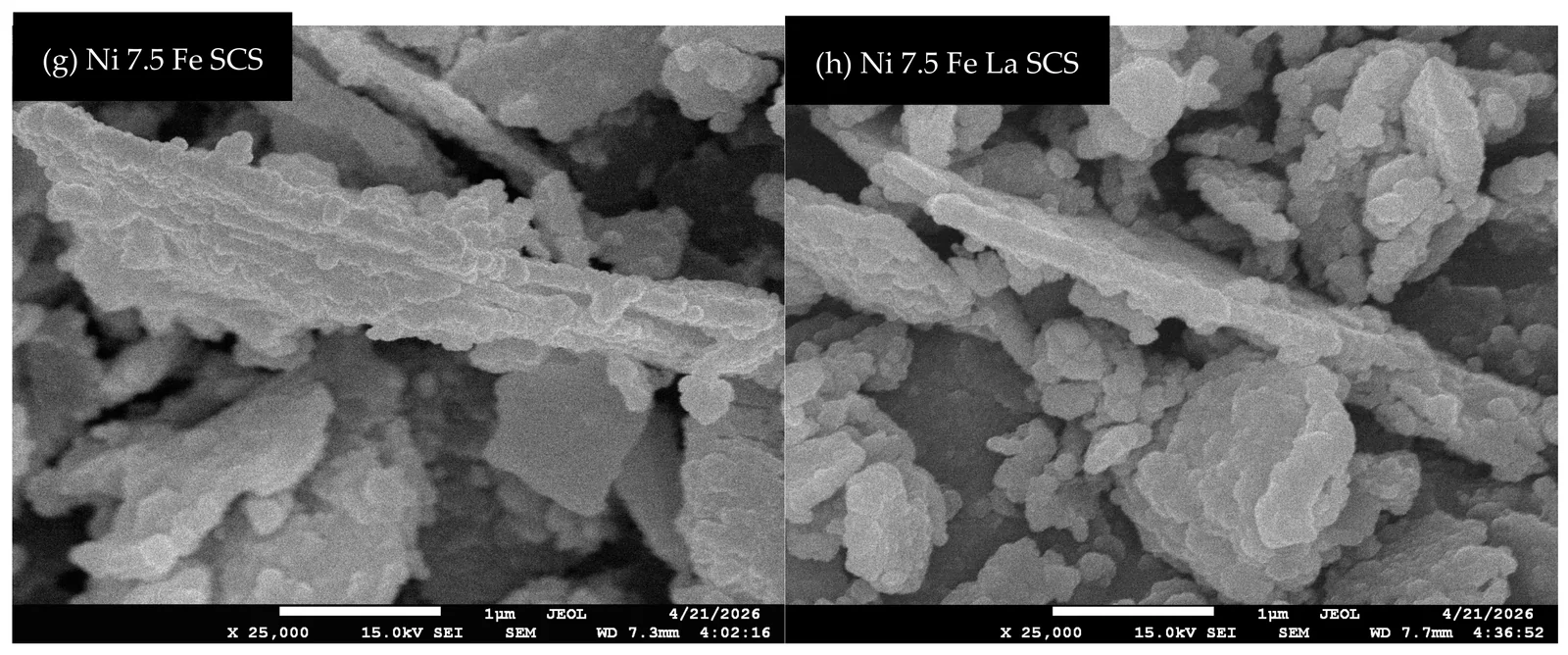
SEM images of Ni-based catalysts prepared by SCS: NiFe catalysts (**a**,**c**,**e**,**g**) and NiFe catalysts promoted with La (**b**,**d**,**f**,**h**).

The morphology of the samples was investigated by SEM. As shown in Figure 3, the SCS-derived catalysts exhibited an irregular morphology with pronounced agglomeration. The samples were composed of fragmented plate-like aluminosilicate particles, characteristic of kaolin-derived support, and smaller oxide aggregates distributed over their surface. The characteristic layered structure of kaolin was partially disrupted, which may be attributed to the rapid combustion process and intensive gas evolution during SCS. The incorporation of Fe did not lead to a complete reconstruction of the morphology. However, Fe-containing samples showed a higher contribution of fine surface aggregates, especially at increased Fe loading.

**Figure 4:**
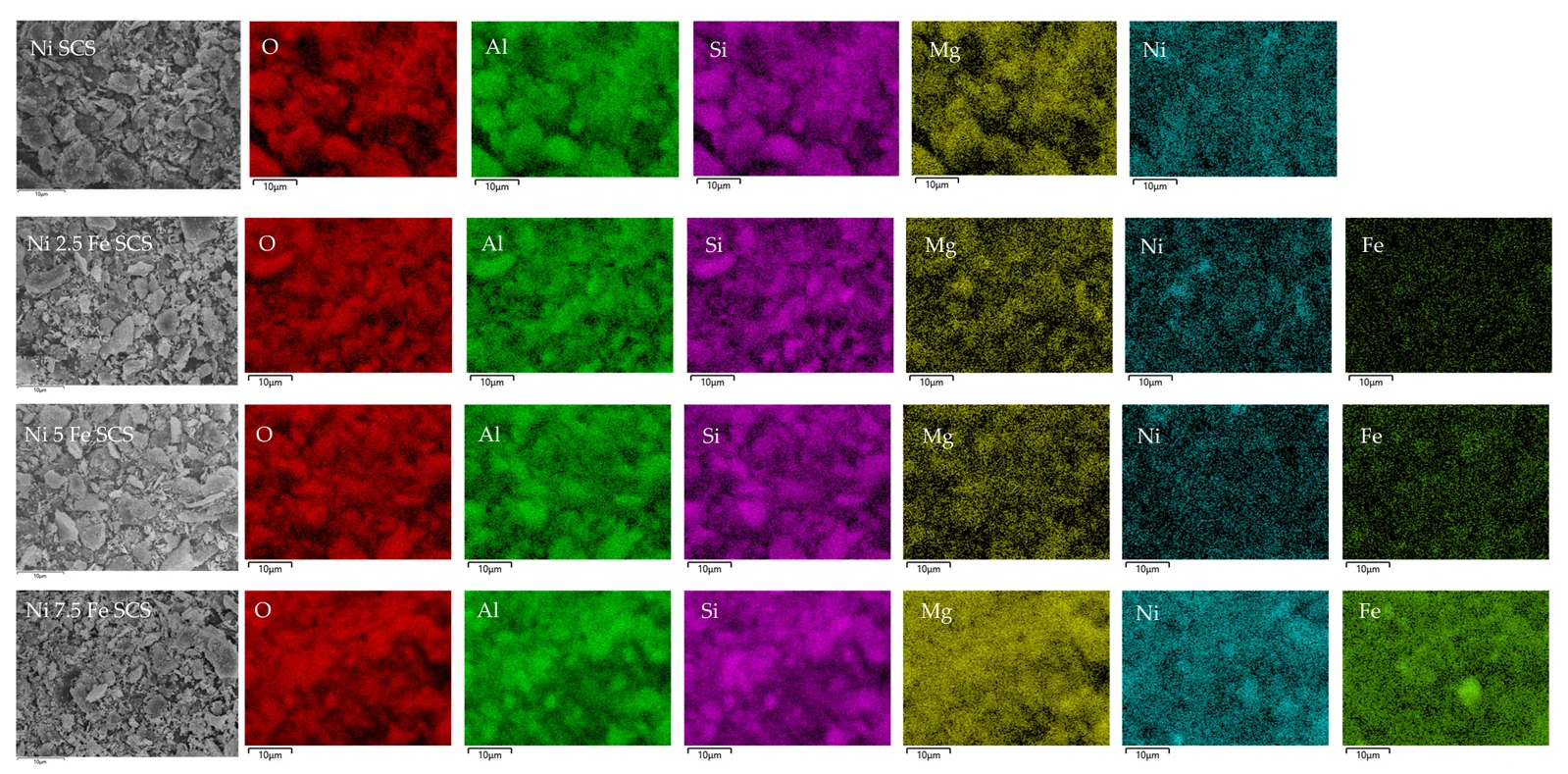
EDS elemental mappings showing the distribution of Ni and Fe in the reduced catalysts.

**Figure 5:**
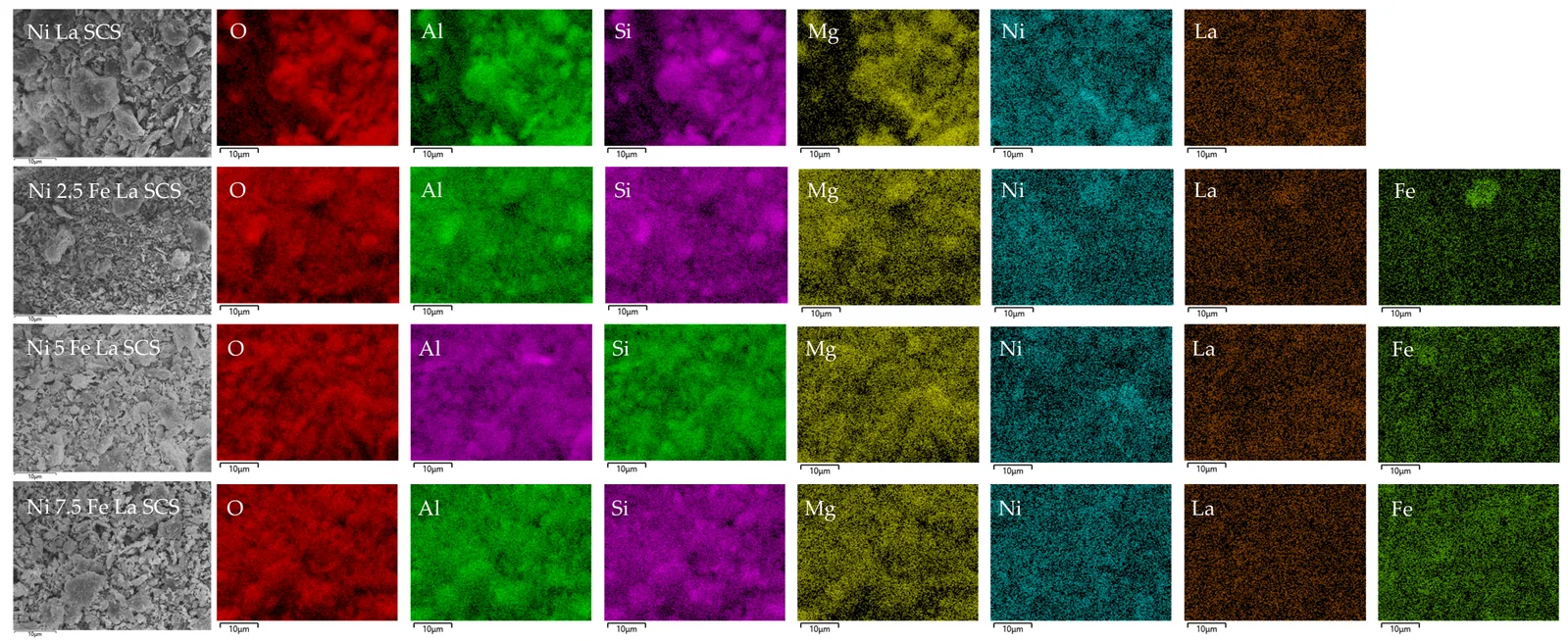
EDS elemental mappings showing the distribution of Ni, Fe, and La in the reduced catalysts.

As shown in Figure 4 and Figure 5, EDS elemental mapping showed that Ni was well-distributed over the surface of the aluminosilicate support in all samples. The maps of Fe-containing catalysts confirmed the presence of iron over the analyzed areas, although slight local enrichment of Fe was observed for the Ni7.5Fe_SCS sample. This suggests partial aggregation of iron-containing species. In the La-promoted series, lanthanum was detected across the catalyst surface, indicating effective introduction of the promoter without the formation of large La-rich aggregates. Overall, SEM/EDS results confirmed the successful incorporation of Ni, Fe, and La species while preserving the fragmented, plate-like morphology of the kaolin-derived support.

#### 2.1.2. Textural Properties

**Figure 6:**
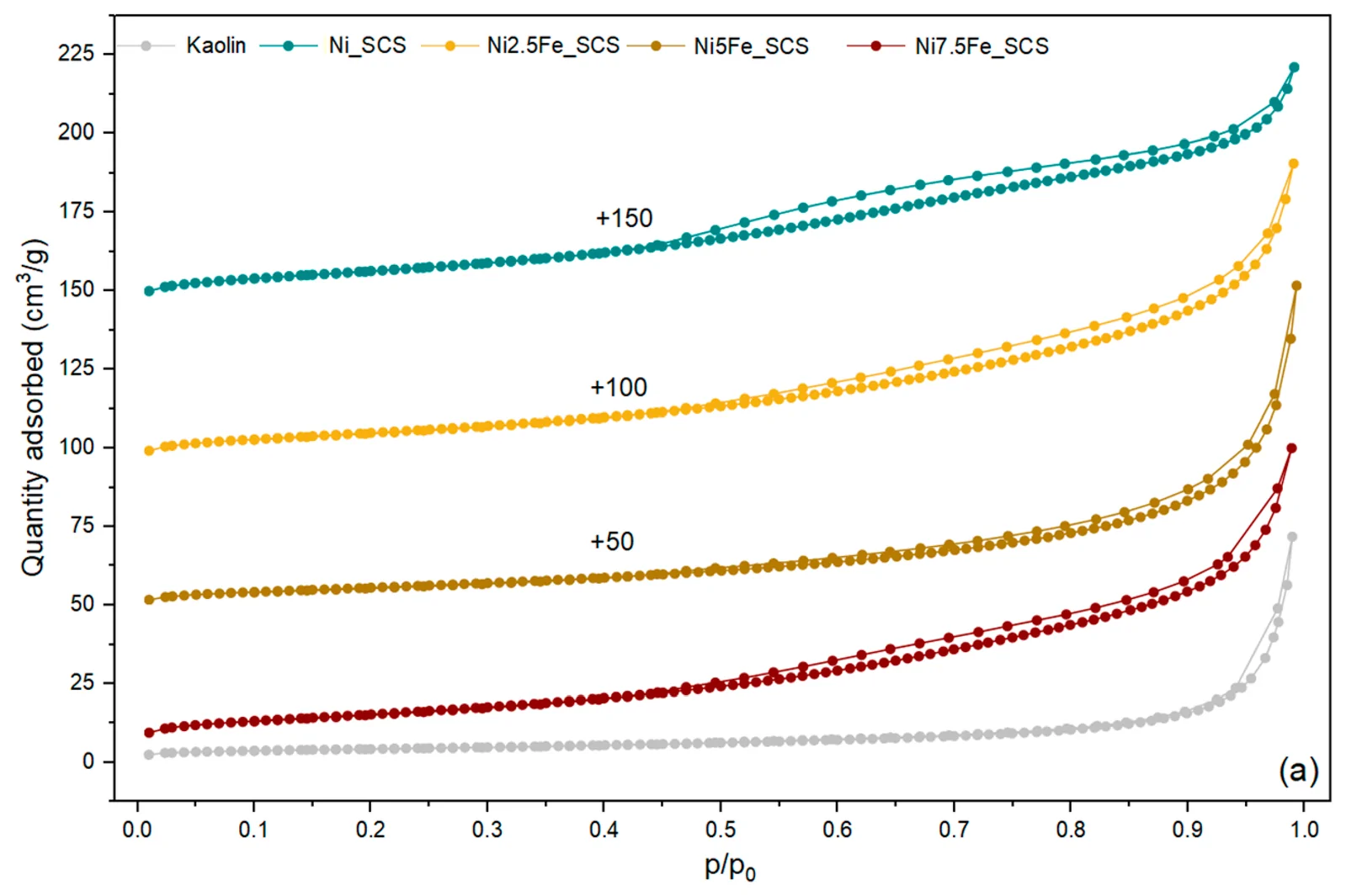

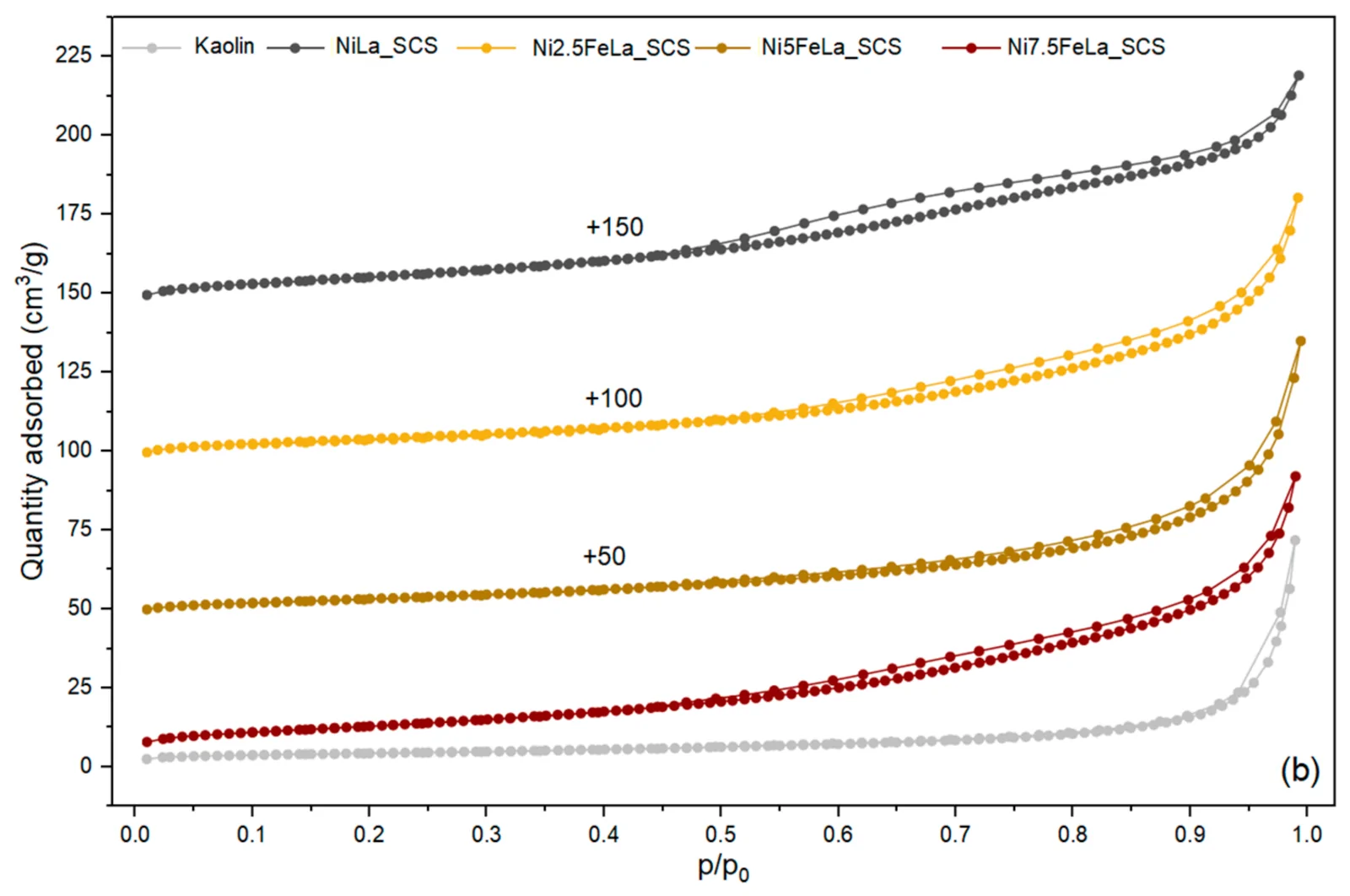
N_2_ adsorption–desorption isotherms obtained for non-modified kaolin, (**a**) NiFe catalysts, and (**b**) NiFeLa catalysts (for better visibility, the isotherms were shifted by the values given in the figures).

Nitrogen adsorption–desorption isotherms of the support (kaolin), the investigated catalysts, and their La-promoted counterparts are presented in Figure 6a,b, respectively. The adsorption–desorption branch of natural kaolin shows features close to type III, according to the IUPAC classification, as reflected by their concave shape and relatively low nitrogen uptake in the intermediate *p*/*p*_0_ region [48]. The weak hysteresis observed at high relative pressures, resembling an H3-type loop, can be associated with slit-shaped voids formed between aggregated plate-like particles. Its low intensity indicates limited capillary condensation, although the possible co-existence of mesoporosity and larger interparticle voids should be taken into account [42]. In contrast, the isotherms obtained for the catalysts exhibit characteristics typical of mesoporous materials. All samples show a gradual increase in nitrogen uptake at low and intermediate relative pressures, followed by a pronounced rise at high relative pressures (*p*/*p*_0_ > 0.85), indicating capillary condensation within mesopores [49]. According to the IUPAC classification [48], the isotherms can be classified as type IV(a), with a H3 hysteresis loop, indicating the presence of mesopores associated mainly with slit-shaped interparticle voids. Importantly, the course of the isotherms was similar for all catalysts, suggesting that the pore structure remains largely unchanged regardless of iron loading and the presence of La. Additionally, the absence of a sharp N_2_ uptake at low *p*/*p*_0_ values suggests that microporosity is not the dominant textural feature of the investigated samples [50].

**Figure 7:**
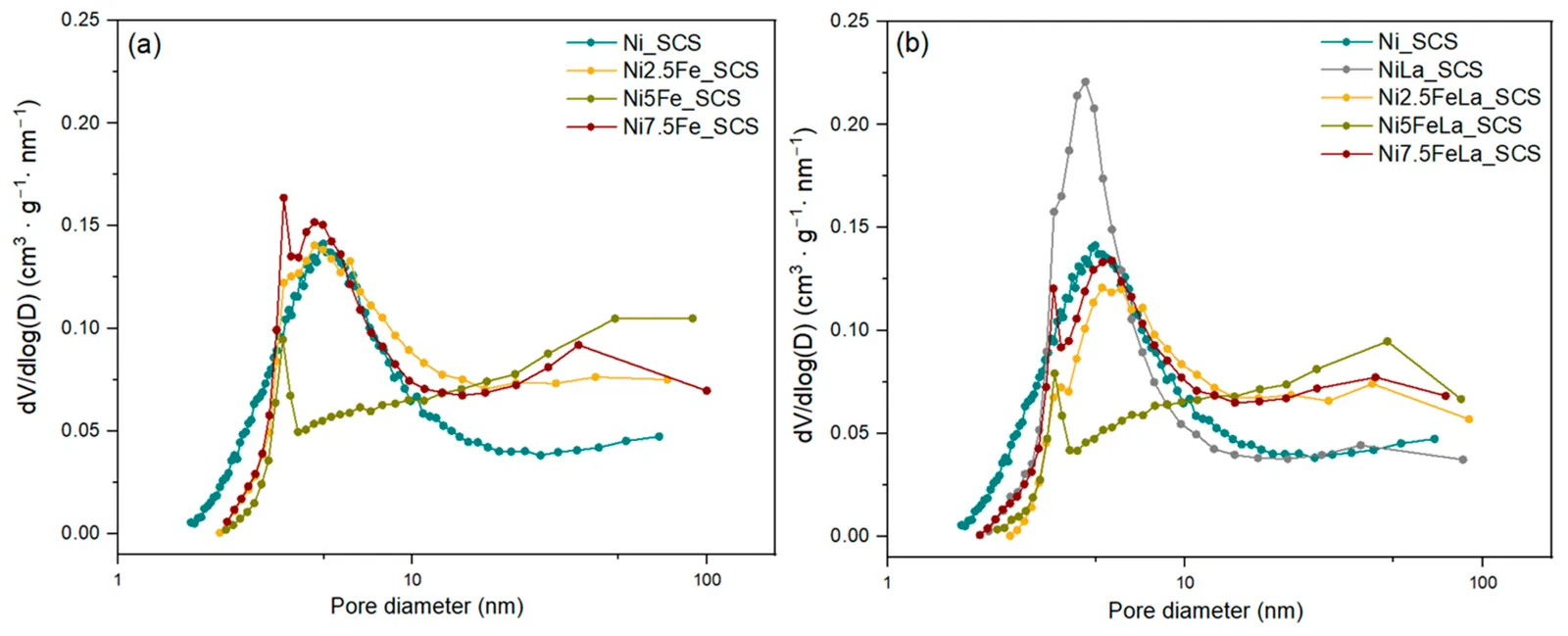
Pore size distribution curves for (**a**) NiFe catalysts and (**b**) NiFeLa catalysts.

The textural parameters of the samples are collected in Table 3. The materials are predominantly mesoporous, with average pore diameters ranging from 9 to 18 nm. The contribution of microporosity is low, indicating that nitrogen adsorption is mainly controlled by multilayer adsorption and pore condensation [51]. In the non-promoted series, the introduction of Fe initially decreased the BET surface area from 59 m^2^/g for Ni_SCS to 37 m^2^/g for Ni5Fe_SCS, accompanied by an increase in the average pore diameter from 9 to 18 nm. Further increasing the Fe content to 7.5 wt.% restored the BET surface area to 55 m^2^/g and decreased the average pore diameter to 11 nm. This non-monotonic behavior may be related to changes in the combustion process caused by the increasing Fe precursor content. Although the fuel-to-oxidized equivalence ratio was kept constant at *φ* = 1 for all catalysts, increasing the Fe precursor content changes the composition and redox characteristics of the nitrate precursor mixture. This may affect the combustion process and consequently, the morphology and textural properties of the resulting oxide [52]. The changes in *S_BET_* were mainly associated with the external surface area, while microporosity remained negligible. Therefore, the lower surface area and larger pore diameter observed for Ni5Fe_SCS may reflect differences in the combustion-derived porous structure at the intermediate Fe content. Further Fe addition, on the other hand, may promote a different structural development. Since the combustion process was not monitored directly, this explanation should be considered as a possible interpretation rather than a definitive mechanism. A similar non-monotonic trend in BET surface area and pore diameter was also observed for the La-promoted samples. The BJH pore size distributions provide further insight into the textural changes observed from the adsorption measurements. As shown in Figure 7, all investigated catalysts exhibit broad pore size distributions, with the main contribution located in the range of approximately 3.6–70 nm. This indicates that the catalysts show mesoporous-macroporous structures, with high number of mesopores, which promote the mass transfer and diffusion of CO_2_ [53,54]. Importantly, for Ni5Fe_SCS, the distribution is particularly broad and extends toward larger pore diameters, which may be associated with larger interparticle voids rather than a distinct population of 48 nm pores. The differences in the average pore diameters listed in Table 3 do not reflect a proportion shift in the dominant pore population toward larger sizes. For example, although Ni5Fe_SCS exhibits the highest average pore diameter (18 nm), its BJH distribution is still dominated by pores of approximately 3.5–5.0 nm. This relatively high average value can therefore be attributed to the broad pore size distribution and contribution of larger pores and/or interparticle voids. The non-monotonic changes in the BJH distributions with increasing Fe content, particularly the broader distribution for Ni5Fe_SCS and the more pronounced small-mesopore contribution for Ni7.5Fe_SCS, are consistent with the changes in BET surface area and average pore diameter, indicating that Fe incorporation modifies the development and distribution of the porous structure, rather than systematically enlarging the dominant mesopores.

As evidenced by the N_2_ sorption branches, the introduction of La does not significantly alter the overall shape of the isotherms, indicating that the predominantly mesoporous character is preserved. However, the textural parameters show some variation upon La addition. In particular, Ni5FeLa_SCS exhibits the highest micropore contribution (*S_micro_* = 9 m^2^/g and *V_micro_* = 0.004 cm^3^/g), suggesting a slightly greater development or preservation of smaller pores. A slight decrease in BET surface area after La impregnation may be associated with partial coverage of the accessible surface by La-containing species or with minor changes in pore accessibility. However, the available textural data do not provide direct evidence for pore blocking. Generally, La impregnation causes only moderate changes in the textural properties, while the predominantly mesoporous character and the fundamental adsorption mechanism remain unchanged. Additionally, as evidenced in Figure 7, the overall mesoporous character of the La-impregnated catalysts is preserved, with moderate changes in the shape and intensity of the BJH distributions. Compared with Ni_SCS, NiLa_SCS shows a slight shift in the main contribution toward smaller pore diameters, from approximately 5 to 4.7 nm. Ni2.5FeLa_SCS exhibits a broader distribution with a pronounced contribution in the range of approximately 3.6–8 nm, whereas Ni5FeLa_SCS and Ni7.5FeLa_SCS show their main contributions at approximately 5.7 and 5.3 nm, respectively. Similarly to its non-promoted counterpart, the additional contribution observed at approximately 48 nm for Ni5FeLa_SCS may be associated with larger interparticle voids rather than a distinct population of mesopores.

#### 2.1.3. Reducibility of the Catalysts

**Figure 8:**
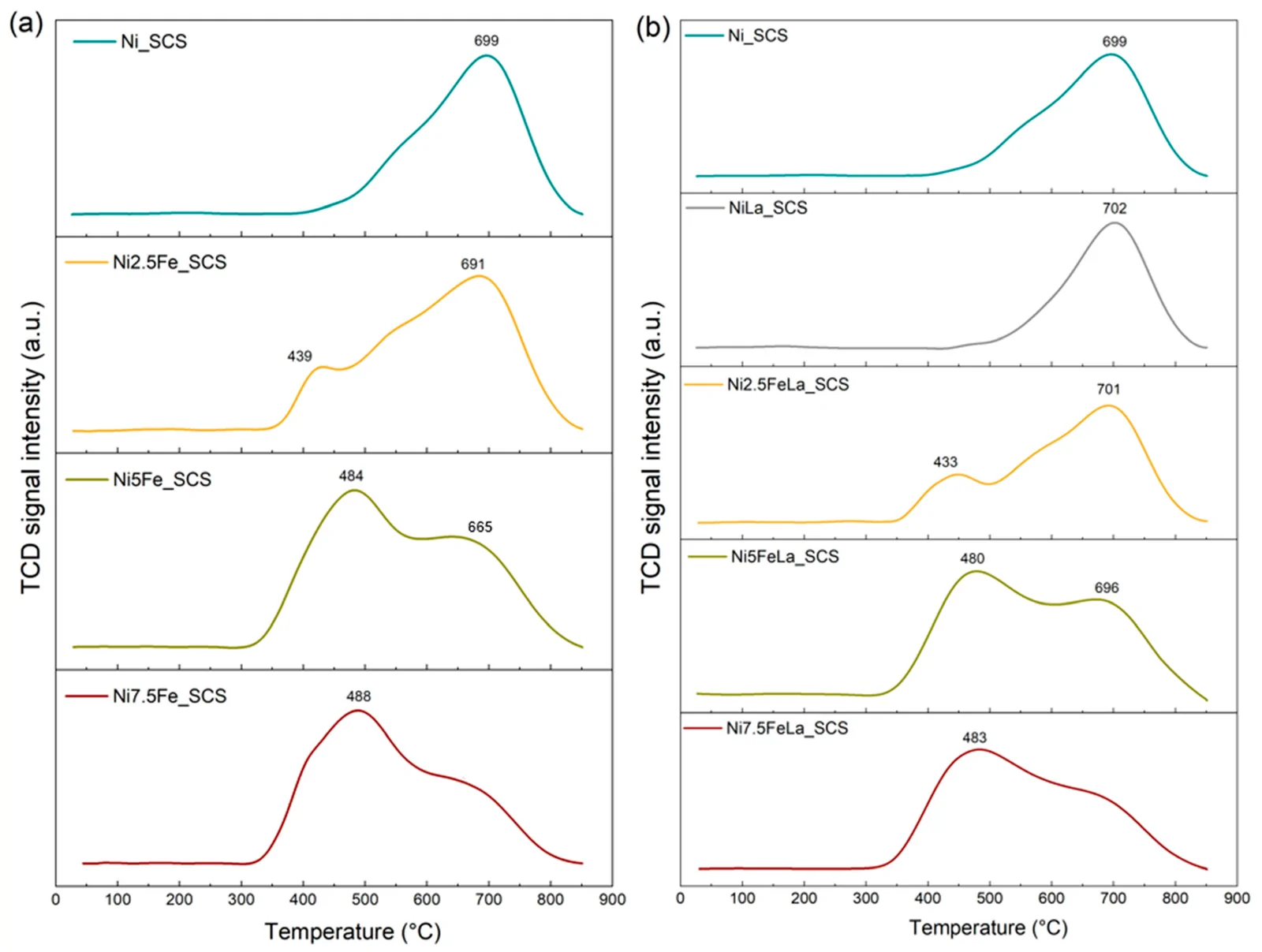
H_2_-TPR profiles obtained for (**a**) NiFe catalysts and (**b**) NiFe catalysts promoted with La.

Since hydrogen plays a key role in the activation of CO_2_ methanation catalysts prior to the reaction, the ability of the catalyst to react with H_2_ is a crucial factor in the process. Reducibility of the catalysts was evaluated by H_2_-TPR to assess the effects of Fe loading and La addition. Typically, the combustion synthesis route may additionally promote intimate contact between the oxide phases and improve metal dispersion, facilitating the reduction in active species [55]. The TPR profiles are presented in Figure 8a,b for NiFe and NiFeLa, respectively. The total consumption of H_2_ detected during H_2_-TPR is collected in Table 4, whereas the deconvoluted temperature peaks are presented in Table 4. In general, the curves presented in Figure 8 can be divided into three main regions: within 400–500 °C (bulk NiO), 500–600 °C (NiO strongly interacting with the support, so-called fixed NiO), and 600–800 °C (nickel aluminate or nickel silicate species reducible only at high temperatures) [56]. Interestingly, the samples did not show any significant reduction features below 400 °C, assigned to highly dispersed nickel oxide species. In the case of Ni_SCS, one sharp reduction peak centered at approximately 700 °C was observed. Thus, the majority of nickel species in the monometallic sample were strongly incorporated into a mixed oxide matrix with a periclase-like structure (Mg(Al,Ni)O). The high-temperature reduction feature may additionally suggest the formation of spinel-like phases, such as (Ni,Mg)Al_2_O_4_ [57]. Moreover, these species could also exist in the form of nickel aluminate or nickel silicate, strongly interacting with the aluminosilicate support [56]. Overall, the Ni_SCS catalyst exhibited limited reducibility due to strong metal–support interactions, reflected by its moderate H_2_ consumption of 2.60 mmol H_2_ per g of the catalyst.

As shown in Figure 8a, Fe addition introduced new reduction features and shifted the reduction temperatures toward lower values, while the total H_2_ consumption strongly depended on Fe loading. The earlier onset of H_2_ consumption was most likely associated with the reduction of Fe^3+^ to Fe^2+^, in agreement with Hoang Ho et al. [29]. At 2.5 wt.% Fe, the total H_2_ consumption decreased to 1.83 mmol H_2_ per g of the catalyst, whereas further Fe addition increased it to 3.87 and 3.93 mmol H_2_ per g for Ni5Fe_SCS and Ni7.5Fe_SCS, respectively. The peaks centered at 439 and 446 °C for Ni2.5Fe_SCS, 484 °C for Ni5Fe_SCS, and 488 °C for Ni7.5Fe_SCS were assigned primarily to the reduction in relatively weakly interacting NiO species deposited on the clay surface [56]. Although the contribution from Fe_2_O_3_→ Fe_3_O_4_ reduction cannot be completely excluded in this temperature range [58], thermodynamic calculations indicate that further reduction of iron oxides is favored above 700 °C [59]. Thus, the high-temperature features are more likely associated with strongly interacting Ni-containing mixed oxides and/or spinel-like phases. The distribution of reducible species was strongly affected by Fe loading. Ni2.5Fe_SCS exhibited the lowest reduction temperatures in the 400–500 °C range, although these features contributed less to the overall reduction due to the larger fraction of high-temperature features. Increasing Fe loading also shifted the high-temperature peak from approximately 700 to 665 °C, which was tentatively assigned to the reduction in the hercynite phase (FeAl_2_O_4_) [32]. The increased H_2_ consumption for Ni5Fe_SCS and Ni7.5Fe_SCS indicates the formation of additional Ni-Fe oxide species. In general, the observed shifts in the TPR peaks with increasing Fe loading can be attributed to changes in the interaction between the Ni and Fe species. An increase in Fe content may alter the local environment of the Ni- and Fe-containing oxide species, as well as the strength of their interaction with the support.

Figure 8b presents the H_2_-TPR profiles of the La-promoted catalysts. Overall, the effect of lanthanum was strongly dependent on Fe content. The total hydrogen consumption of the La-containing samples followed a trend similar to that observed for the corresponding La-free materials. Based on the observations of Hoang Ho et al. [29], the reduction temperature of nickel species is generally not affected by the addition of La. However, in our case, the effect of lanthanum was particularly pronounced for the iron-free sample and the catalyst containing 2.5 wt.% Fe. First, the introduction of La led to the disappearance of the small reduction peaks at 455 and 560 °C observed for Ni_SCS. Moreover, the H_2_ consumption associated with the reduction peak centered at 700 °C for NiLa_SCS was the highest among all investigated samples. Both the position and the intensity of this peak indicate the strongest interaction between the reducible species and the support for this catalyst. The introduction of La did not significantly affect the reducibility of the sample with 2.5 wt.% Fe. In contrast, for the samples with higher Fe loading, the reduction peaks at 665 °C (Ni5Fe_SCS) and 675 °C (Ni7.5Fe_SCS) were shifted towards 696 and 692 °C, respectively, after La promotion. This behavior may indicate enhanced metal–support interactions and/or stabilization of dispersed NiO species by lanthanum at increased Fe content. Additionally, lanthanum incorporation resulted in a slight decrease in the total H_2_ consumption for Ni5FeLa_SCS and Ni7.5FeLa_SCS. This effect may be associated with the stabilization of nickel-containing oxide species and strengthening of metal–support interactions, which makes a fraction of the active phase less accessible to reduction.

#### 2.1.4. Basicity of the Catalysts

**Figure 9:**
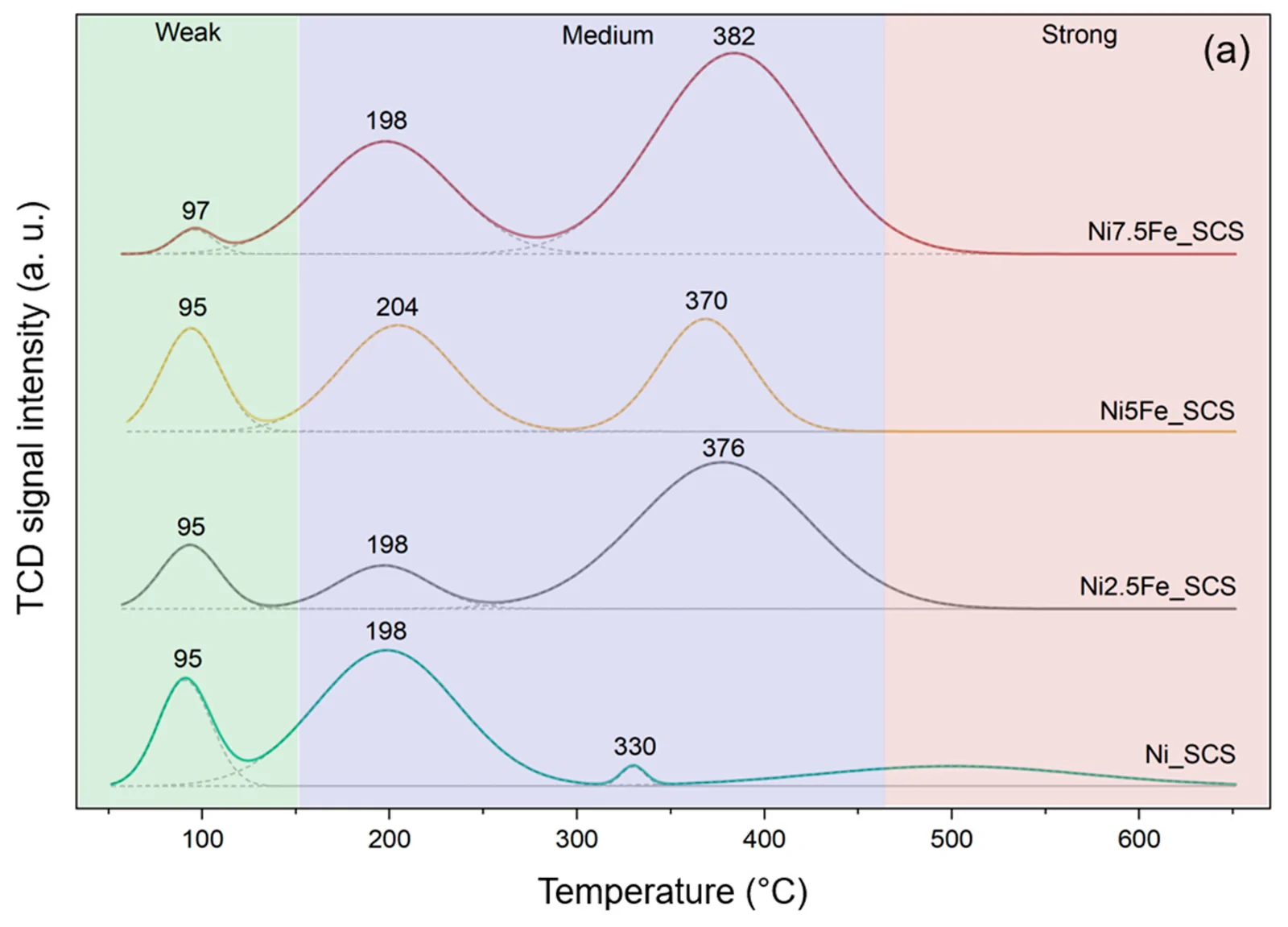

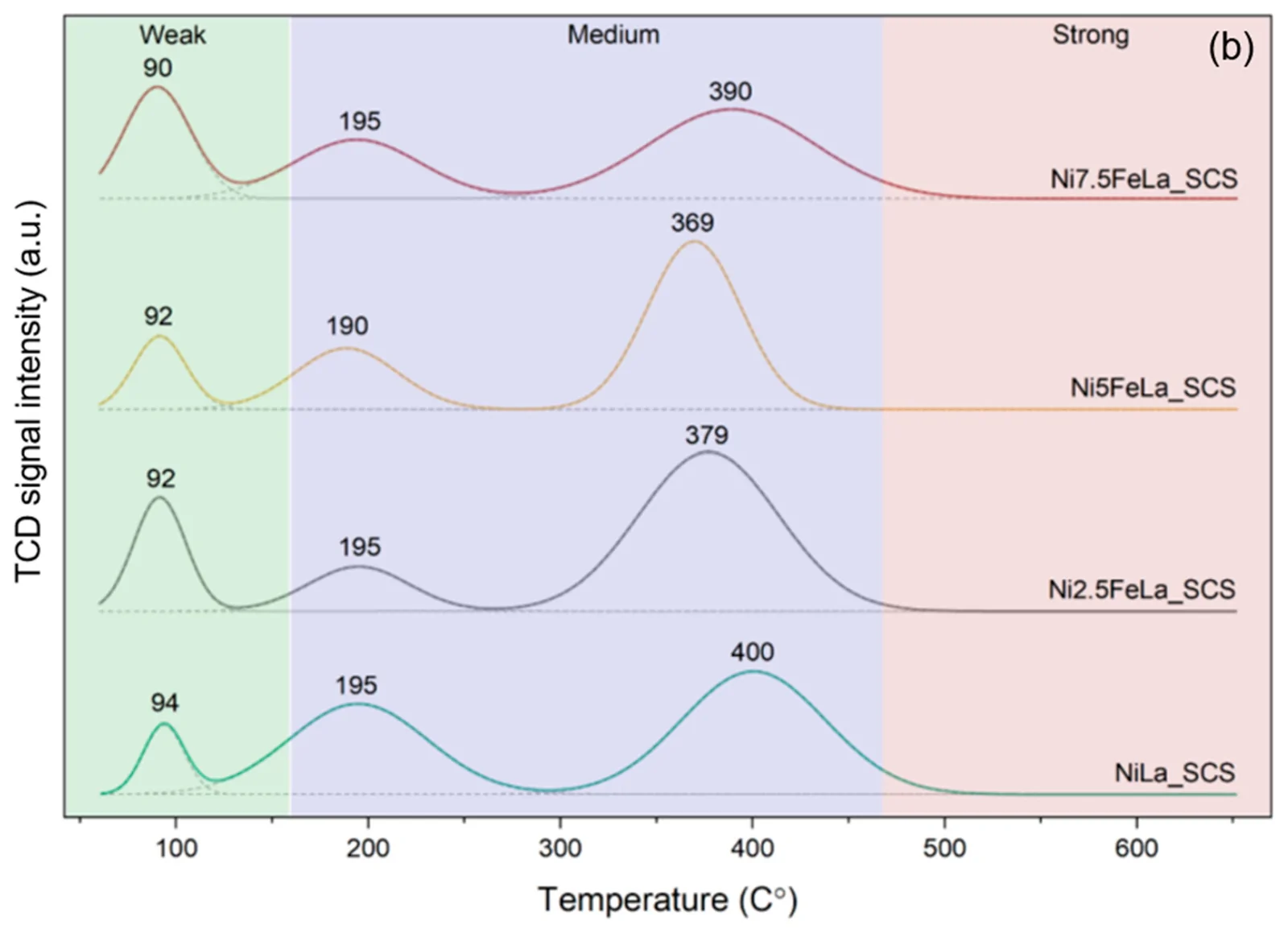
CO_2_-TPD profiles obtained for (**a**) NiFe catalysts and (**b**) NiFe catalysts promoted with La.

The strength and distribution of the surface basic sites on the reduced catalysts were investigated by CO_2_-TPD, which provides information on the interaction of CO_2_ with the catalyst surface. Since carbon dioxide is one of the reactants in the methanation reaction, the CO_2_-TPD profiles also provide valuable information regarding its adsorption and activation [60,61]. Typically, the desorption signals are classified into three temperature ranges, corresponding to weak (below 150 °C), medium (150–450 °C), and strong basic sites (above 450 °C) [62]. The low-temperature desorption peaks are generally attributed to weakly bound CO_2_ species adsorbed on surface hydroxyl groups (Brönsted basic sites). The medium-strength basic sites are mainly assigned to metal-oxygen pairs, exhibiting Lewis basic character. High-temperature desorption peaks are commonly related to low-coordinated surface oxygen (O^2−^) [63]. CO_2_-TPD profiles recorded for the Ni-Fe samples are presented in Figure 9a, whereas the results for their La-promoted counterparts are displayed in Figure 9b. All catalysts exhibited only weak and medium-strength basic sites. This effect may be beneficial for the methanation process, since excessively strong basic sites hinder CO_2_ conversion due to the formation of stable carbonate species that are difficult to hydrogenate [64]. For Ni_SCS three well-resolved desorption peaks were detected: at 95, 198, and 330 °C, which indicates the presence of weak and medium-strength basic sites, typical of Ni-based catalysts [61]. The introduction of iron shifted the medium-temperature desorption peaks towards higher temperatures, indicating stronger interaction between CO_2_ and the basic sites, particularly for Ni7.5Fe_SCS (from 330 to 382 °C). At the same time, the intensity of the peak at 95–97 °C significantly decreased for this sample. Thus, the presence of Fe reduces the contribution of weak basic sites, along with an increase in the concentration of stronger sites. This behavior may arise from several factors, such as incomplete reduction of Fe^3+^ [65] and increased binding energy of the moderate basic sites, as well as changes in the environment of surface oxygen atoms and modified interactions between the active phase and support matrix [59,66]. The results for La-promoted catalysts revealed that, similarly to Fe, individual addition of La increased basicity of Ni-catalyst, particularly at higher desorption temperatures. The shift in the medium-temperature desorption peak towards higher temperatures, together with the considerably increased amount of desorbed CO_2_, may be associated with the presence of basic La-containing species. These findings are in good agreement with previous reports [27,67]. Interestingly, the shift in the desorption peak towards higher temperatures was not observed for Ni-Fe samples after promotion with La. Therefore, the interaction between Fe and La cannot be regarded as a simple additive behavior. This observation suggests that the simultaneous presence of Fe and La modifies the nature of the basic sites rather than simply increasing their strength.

#### 2.1.5. Surface Chemical State of the Catalysts

**Figure 10:**
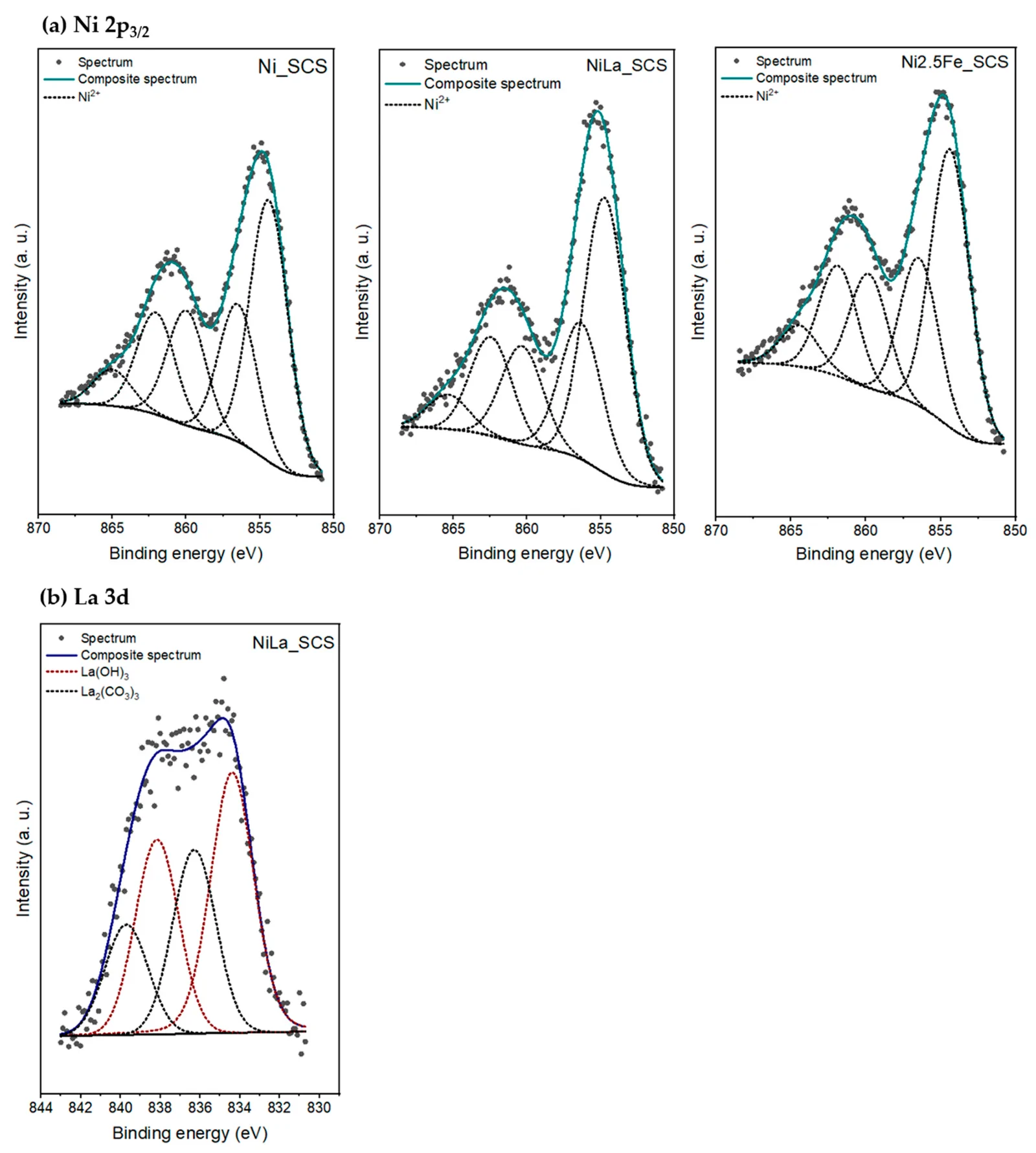

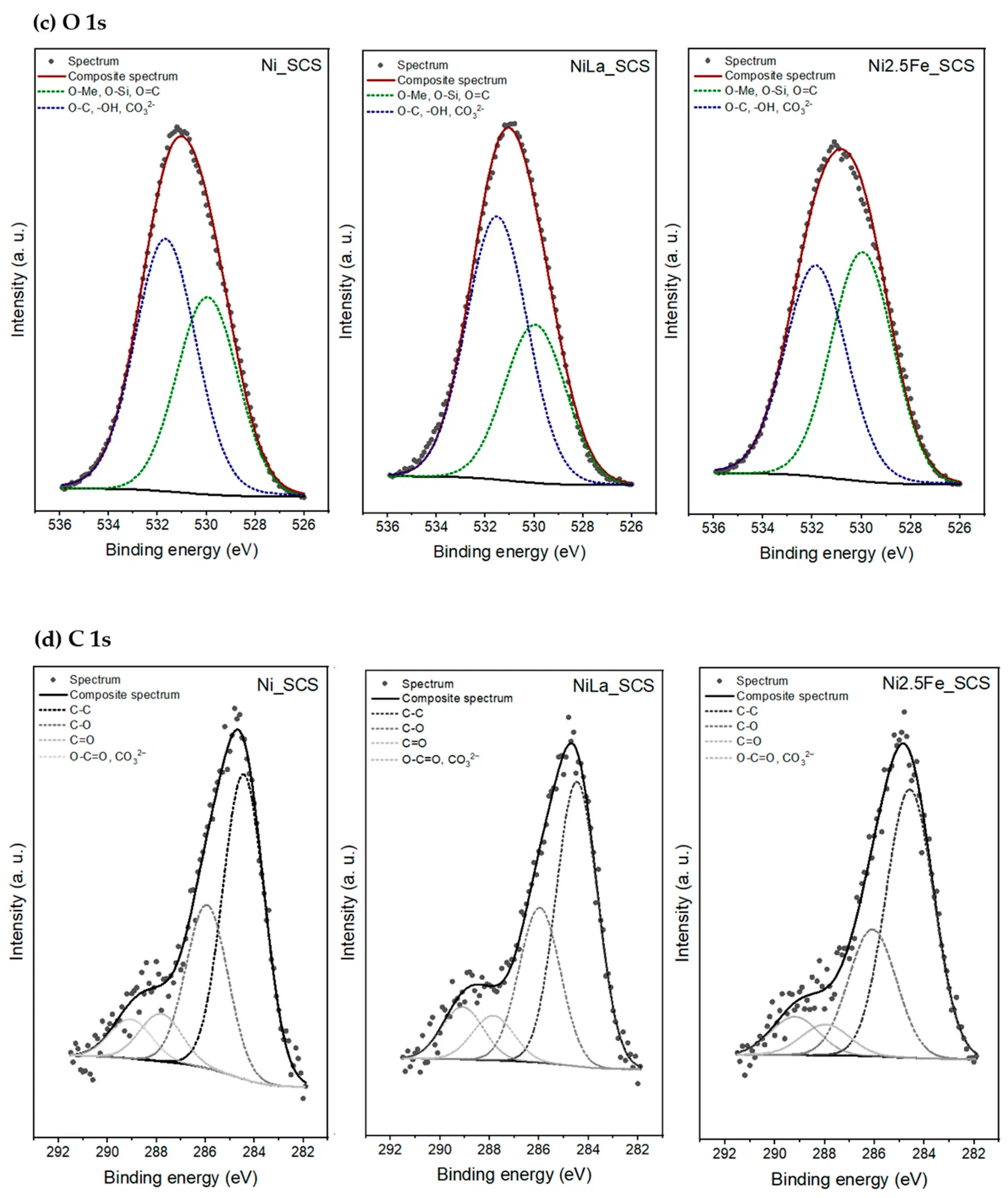
XPS spectra for the reduced Ni_SCS, NiLa_SCS, and Ni2.5Fe_SCS: (**a**) Ni 2p region, (**b**) La 3d region, (**c**) O 1s region, and (**d**) C 1s region.

The XPS technique was employed to further clarify the surface chemical states of nickel in the selected catalysts. Furthermore, it enabled the evaluation of how Fe or La promotion affects the oxidation states and elemental distribution at the catalyst surface. The XPS analysis was performed for a representative subset of samples, specifically Ni_SCS, NiLa_SCS, and Ni2.5Fe_SCS. The choice was driven not only by their superior performance in CO_2_ methanation, but also by the need to systematically isolate and compare the individual electronic effects induced by Fe or La promotion compared to the unpromoted Ni matrix. The binding energy values along with the surface elemental composition are summarized in Table 5, whereas the recorded spectra for the reduced catalysts are presented in Figure 10. The survey X-ray photoelectron spectra of the investigated catalysts are presented in Figure S4.

The Ni 2p_3/2_ spectra, presented in Figure 10a, were fitted with five lines, with the first line centered at 854.7 eV, which may indicate the presence of Ni^2+^ type compounds like NiO or NiOOH [68]. The four lines within the energy range of 857.0–866.0 eV are due to the multiplet splitting phenomena and additionally prove the Ni^2+^ oxidation state. Importantly, the lack of metallic nickel on the samples, with simultaneous existence of nickel oxide and nickel hydroxide my result from their contact with atmospheric air, which contains oxygen and water vapor that caused oxidation of Ni^0^ species. Therefore, the absence of metallic Ni^0^ in the XPS spectra is attributed to the spontaneous surface passivation/re-oxidation prior to ex situ analysis, whereas the bulk reduction was fully confirmed by XRD measurements. Additionally, the absence of the peak at 875.0 eV confirms the absence of NiAl_2_O_4_ spinel-type oxides on the surface of the analyzed samples [11]. Additionally, no measurable differences in the binding energy, spectral line shape, fitted multiplet and satellite structure, or relative intensities were observed after the introduction of Fe or La. Thus, within the experimental uncertainty of the XPS measurements, the promoters did not cause a detectable alteration of the surface chemical or electronic state of Ni.

The XPS spectrum of NiLa_SCS in the region characteristic for La 3d_5/2_ (Figure 10b) displayed a broad feature (831.0–843.0 eV), which was deconvoluted into characteristic doublets, assigned to La(OH)_3_ (834.3 eV) [69] and La_2_(CO_3_)_3_ (836.1 eV) [69] and La_2_(CO_3_)_3_ (836.1 eV) [70]. According to the literature [71,72], the formation of lanthanum carbonate species promotes strong metal–support interactions and enhances redox capacity of Ni-catalysts, thereby facilitating CO_2_ activation and boosting methanation activity. No Fe-related signal was discernible for the Ni2.5Fe_SCS sample under the applied XPS acquisition conditions, indicating that the Fe concentration within the analyzed near-surface region was below the effective detection limit. This observation may result from surface depletion, an inhomogeneous or predominantly subsurface Fe distribution, attenuation by a Ni-rich surface layer, or instrumental detection limitations; therefore, it cannot be regarded as unambiguous evidence of Fe incorporation into the Ni lattice.

The spectra collected in O 1s (Figure 10c) display a remarkable similarity across all analyzed samples. Deconvolution of the O 1s region revealed two primary oxygen species: the first component centered at 530.0 eV, associated with the surface lattice oxygen (O^2−^) of metal oxides (Al-O, Mg-O, La-O), along with minor contributions from carbonyl O=C bonds. The second line, located at 531.7 eV, corresponds to surface oxygen species in defective metal oxide lattices, siloxane/silicate bonds, surface hydroxyl groups (-OH), and adsorbed moisture [70,73,74].

Deconvolution of the C 1s region in the spectra recorded for the investigated samples yielded four characteristic peaks (Figure 10d). The main peak, centered at 284.8 eV, arises from aliphatic carbon (C-C/C-H) and was used as the internal binding energy reference. The second component, located at 286.3 eV corresponds to single oxygen-bearing species, such as C-O and/or C-OH groups [75,76]. The components at 288.3 eV and 289.4 eV correspond to C=O/O-C-O groups and higher oxidation state carbon species, such as carboxylates (O-C=O) and surface carbonates (CO_3_^2−^), respectively [76].

The Mg 1s spectra (Figure S3a) were fitted with a single component, centered at 1303.2 eV, confirming the presence of Mg^2+^ species within the oxide or hydroxide [77]. The Si 2p region (Figure S3b) exhibited a characteristic doublet structure (with a spin–orbit splitting ΔE p_3/2_ − p_1/2_ = 0.6 eV) and the primary Si 2p_3/2_ peak located at 101.8 eV, pointing to the silicate network of the support [78]. Due to the severe overlap between the Al 2p line with the Ni 3p line, the aluminum chemical state was evaluated using Al 2s region instead (Figure S3c). The Al 2s spectrum was fitted with a single component at a binding energy of 118.8 eV, corresponding to framework Al^3+^ species [79].

The surface elemental composition (in at.%) and binding energies obtained from the XPS analysis of the selected catalysts are summarized in Table 5. The surface concentration of nickel varied depending on the promoter added: Ni_SCS catalyst exhibited a surface nickel content of 9.9 at.%, which increased to 11.8 at.% for Ni2.5Fe_SCS, indicating an enhanced surface exposure of nickel in the presence of iron. Conversely, the addition of lanthanum in NiLa_SCS led to a slight decrease in surface nickel content to 7.3 at.%. The analysis revealed that lanthanum exists in two distinct chemical states on the outermost surface, contributing to a total surface concentration of 0.8 at.%. Specifically, the deconvolution of the La 3d core-level region confirmed that La is present predominantly as La(OH)_3_ (0.5 at.%) alongside surface-bound lanthanum carbonate (0.3 at.%). Therefore, the attenuation of the surface nickel signal can be primarily attributed to partial geometric coverage (shading effect) of the nickel species by the well-dispersed lanthanum hydroxides/carbonates. Notably, the NiLa_SCS catalyst displayed the highest surface concentration of aluminum (12.2 at.%), whereas the surface concentrations of Si and Mg remained similar for all samples.

### 2.2. Catalytic Tests

**Figure 11:**
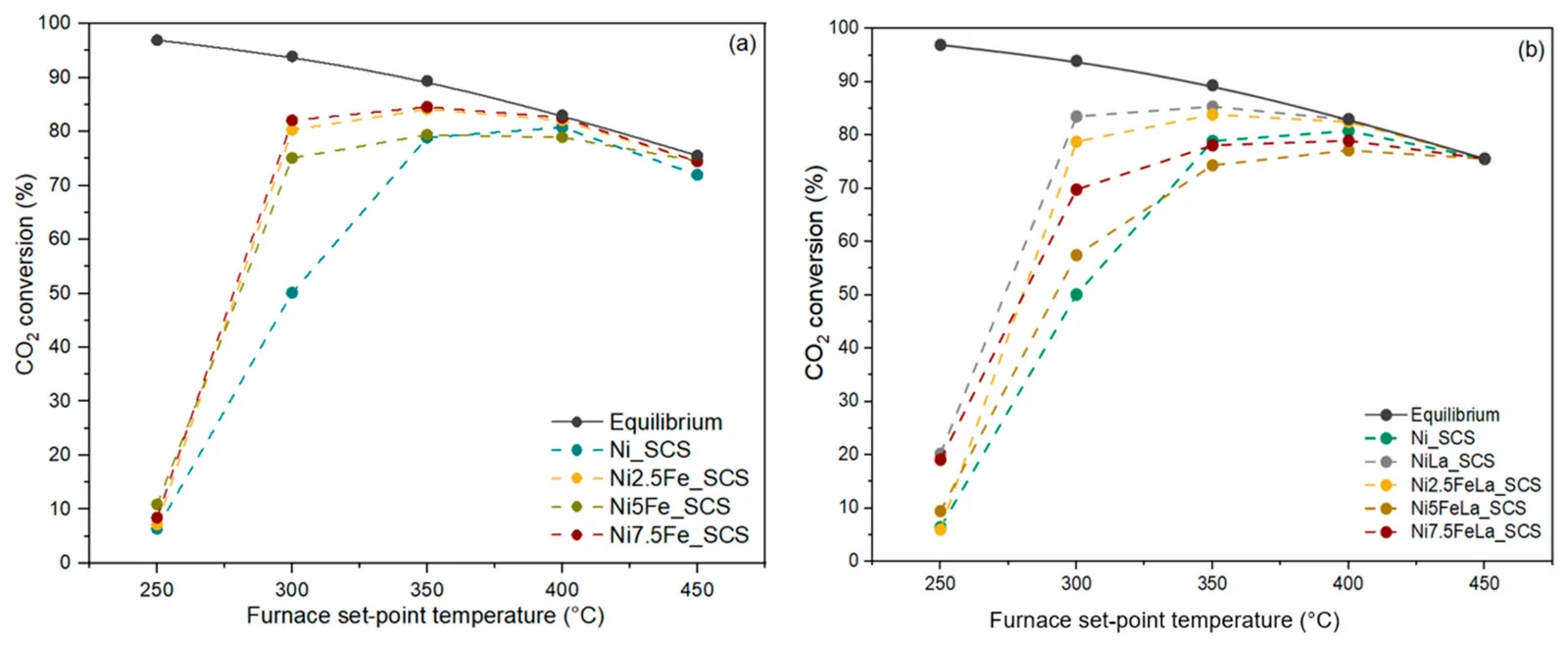
CO_2_ conversion as a function of temperature for (**a**) NiFe catalysts; (**b**) NiFe catalysts promoted with La (reaction conditions: 1 atm; 15 vol.% CO_2_, 60 vol.% H_2_, and 25 vol.% Ar; total flow rate = 100 mL·min^−1^; GHSV = 12,000 h^−1^; catalyst bed volume = 0.5 cm^3^; pretreatment in 5 vol% H_2_/Ar at 600 °C for 1 h).

**Figure 12:**
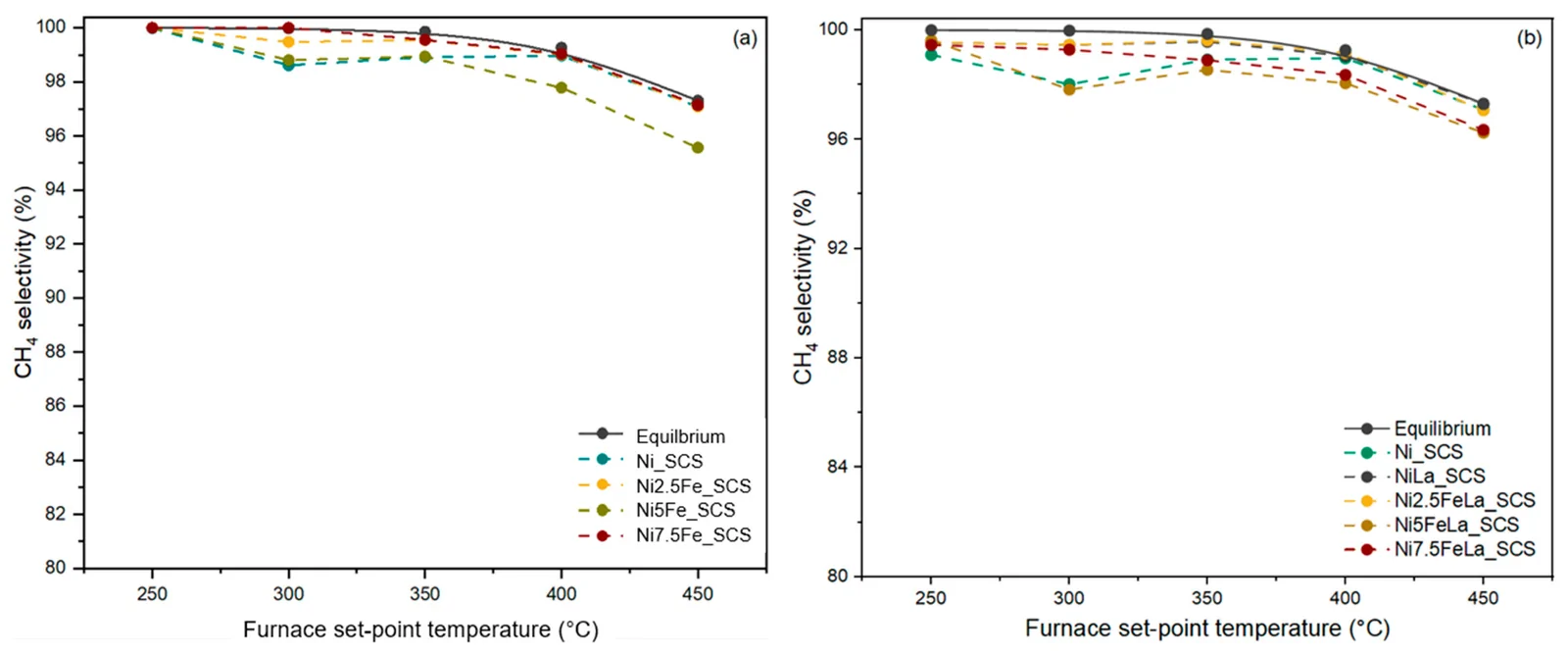
CH_4_ selectivity as a function of temperature for (**a**) NiFe catalysts; (**b**) NiFe catalysts promoted with La (reaction conditions: 1 atm; 15 vol.% CO_2_, 60 vol.% H_2_, and 25 vol.% Ar; total flow rate = 100 mL·min^−1^; GHSV = 12,000 h^−1^; catalyst bed volume = 0.5 cm^3^; pretreatment in 5 vol% H_2_/Ar at 600 °C for 1 h).

**Figure 13:**
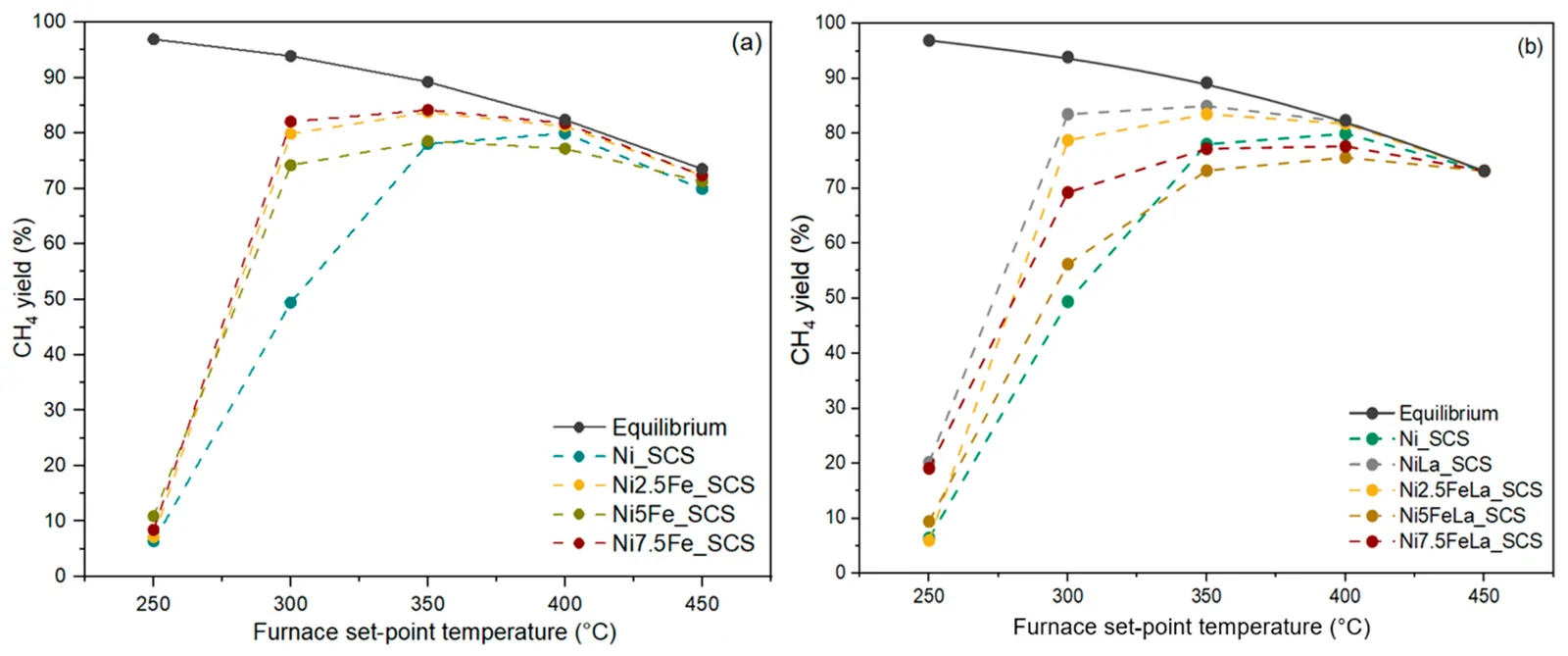
CH_4_ yield as a function of temperature for (**a**) NiFe catalysts; (**b**) NiFe catalysts promoted with La (reaction conditions: 1 atm; 15 vol.% CO_2_, 60 vol.% H_2_, and 25 vol.% Ar; total flow rate = 100 mL·min^−1^; GHSV = 12,000 h^−1^; catalyst bed volume = 0.5 cm^3^; pretreatment in 5 vol% H_2_/Ar at 600 °C for 1 h).

All of the prepared catalysts were tested in CO_2_ methanation. The results obtained in terms of CO_2_ conversion, CH_4_ selectivity, and CH_4_ yield are presented in Figure 11, Figure 12 and Figure 13, respectively. The experimental results were compared with the thermodynamic equilibrium values to assess the contribution of kinetic and thermodynamic limitations. It should be noted that the temperatures presented in Figure 11, Figure 12 and Figure 13 correspond to the furnace set-point temperatures, whereas the actual catalyst-bed temperature was approximately 50 °C higher under the applied reaction conditions. Thus, the apparent sharp increase in CO_2_ conversion between the nominal temperatures of 250 and 300 °C corresponds to an increase in the catalyst temperature from approximately 300 to 350 °C. The approximately 50 °C difference between the furnace set-point and the catalyst-bed temperature was reproducible under the applied reaction conditions and remained consistent throughout the tested temperature range. Therefore, it does not indicate an abrupt temperature excursion occurring specifically in the 250–350 °C range. Nevertheless, since the catalyst temperature was monitored at a specific position within the bed, minor local temperature gradients cannot be completely excluded.

As confirmed by Wang et al. [80], CO_2_ methanation is governed by both thermodynamic and kinetic factors, while the equilibrium CH_4_ yield remains high (>85%) below 400 °C for stoichiometric H_2_/CO_2_ mixtures under near-atmospheric pressure. In the present study, the experimental results approached equilibrium within the nominal temperature range of 350–400 °C for Ni2.5Fe_SCS and Ni7.5Fe_SCS. For the La-promoted catalysts, CO_2_ conversion was close to equilibrium at 350–450 °C for NiLa_SCS and Ni2.5FeLa_SCS, and at 400–450 °C for the remaining samples except for Ni5FeLa_SCS. Furthermore, all Ni-Fe catalysts except Ni5Fe_SCS showed CH_4_ selectivity close to the equilibrium values within the nominal temperature range of 350–450 °C, while among the La-promoted catalysts this was observed for NiLa_SCS and Ni2.5FeLa_SCS.

As shown in Figure 11, at the nominal temperature of 250 °C, the catalysts generally exhibited low CO_2_ conversion, regardless of Fe loading or La promotion. Only NiLa_SCS and Ni7.5FeLa_SCS reached approximately 20% CO_2_ conversion, whereas Ni_SCS exhibited the lowest activity, particularly at 300 °C. A marked increase in conversion was observed with increasing temperature, with the effect of Fe incorporation being most pronounced at 300 °C. The strongest improvement was observed for catalysts containing 2.5 and 7.5 wt.% Fe. At higher temperatures, the differences between the Ni-Fe catalysts became less pronounced, as their CO_2_ conversions approached the thermodynamic equilibrium. These results suggest that Fe incorporation can improve the ability of the catalysts to approach thermodynamic equilibrium, although its loading requires careful optimization. The relatively lower performance of Ni5Fe_SCS may also be related to its specific physicochemical characteristic. The effect of La depends strongly on the catalyst composition. Impregnation with 3 wt.% La substantially improved the performance of Ni_SCS, with NiLa_SCS exhibiting the highest CO_2_ conversion among the investigated catalysts, particularly at low temperature. In contrast, La impregnation generally decreased the activity of the Fe-containing catalysts, with the exception of Ni7.5FeLa_SCS. Thus, no monotonic relationship between Fe loading and catalytic activity was observed. The convergence of the experimental CO_2_ conversions toward the equilibrium values at 400–450 °C indicates that at these temperatures the reaction becomes predominantly limited by thermodynamics, which reduces the differences arising from catalyst composition and intrinsic activity. Consequently, the beneficial effect of catalyst modification is most clearly manifested at lower temperatures.

Figure 12 presents the CH_4_ selectivity of the investigated catalysts. All samples remained highly selective toward CH_4_ over the entire temperature range, with values exceeding 96%. At 250 °C, all Ni-Fe catalysts reached CH_4_ selectivity close to the thermodynamic equilibrium values, followed by a slight decrease with increasing temperature. This decrease was most pronounced for Ni5Fe_SCS. For the La-promoted catalysts, the decrease in CH_4_ selectivity observed at Fe loadings above 2.5 wt.% may indicate an increased contribution of the RWGS reaction and/or the formation of other carbon-containing products. However, since higher hydrocarbons were not specifically analyzed in the present study, their possible contribution cannot be completely excluded. However, this interpretation should be treated with caution, as the decrease in selectivity may also arise from other kinetic or physicochemical effects. Notably, this trend was not observed for NiLa_SCS, suggesting that La alone does not appreciably enhance RWGS under the applied reaction conditions.

The CH_4_ yield obtained over the investigated catalysts is presented in Figure 13. Since all materials exhibited high CH_4_ selectivity throughout the investigated temperature range, the CH_4_ yield closely followed the trends observed in CO_2_ conversion. Accordingly, low CH_4_ yields were obtained at 250 °C, reflecting the limited CO_2_ conversion at this temperature, whereas increasing the temperature resulted in a marked increase in CH_4_ yield. At higher temperatures, the experimental yields approached the thermodynamic equilibrium values, consistent with the observed convergence of CO_2_ conversion and CH_4_ selectivity toward equilibrium.

To enable a more rigorous comparison of the catalytic performance of the investigated catalysts, the CO_2_ conversion rates were normalized to both the catalyst mass and BET surface area. The corresponding mass-normalized and BET surface-area-normalized CO_2_ conversion rates are summarized in Table 6. The highest mass-normalized CO_2_ conversion rate was observed for NiLa_SCS (135.27 mmol·g^−1^·h^−1^), closely followed by Ni2.5FeLa_SCS (135.12 mmol·g^−1^·h^−1^). Considering the negligible difference between these values, the addition of 2.5 wt.% Fe did not significantly affect the mass-normalized activity of the La-promoted catalyst. Further increasing the Fe content resulted in lower mass-normalized CO_2_ conversion rates, although no monotonic relationship between Fe loading and catalytic activity was observed. In the La-free series, the introduction of Fe increased the mass-normalized CO_2_ conversion rate compared with Ni_SCS, with the highest value obtained for Ni2.5Fe_SCS. A further increase in the Fe content led to a decrease in the mass-specific activity; however, all Fe-promoted samples still exhibited higher CO_2_ conversion rates than the unpromoted Ni_SCS catalyst. These results indicate that a low Fe loading was beneficial for the overall catalytic performance, whereas increasing the Fe content did not provide further improvement. A different trend was observed after normalization of the CO_2_ conversion rate to the BET surface area. The highest BET surface-area-normalized CO_2_ conversion rate among all investigated catalysts was obtained for Ni5FeLa_SCS (3.56 mmol·m^−2^·h^−1^). Interestingly, Ni5Fe_SCS also exhibited the highest value within the La-free series (2.53 mmol·m^−2^·h^−1^). Therefore, the addition of 5 wt.% Fe appears to provide the most favorable catalytic activity per unit BET surface area, irrespective of the presence of La. The different trends observed for the mass-normalized and BET surface-area-normalized conversion rates indicate that the catalytic performance cannot be explained solely by differences in the specific surface area. Instead, other factors, such as the nature, density, and accessibility of the active sites, metal dispersion, and interactions between the active components, are likely to play an important role in determining the CO_2_ conversion.

**Figure 14:**
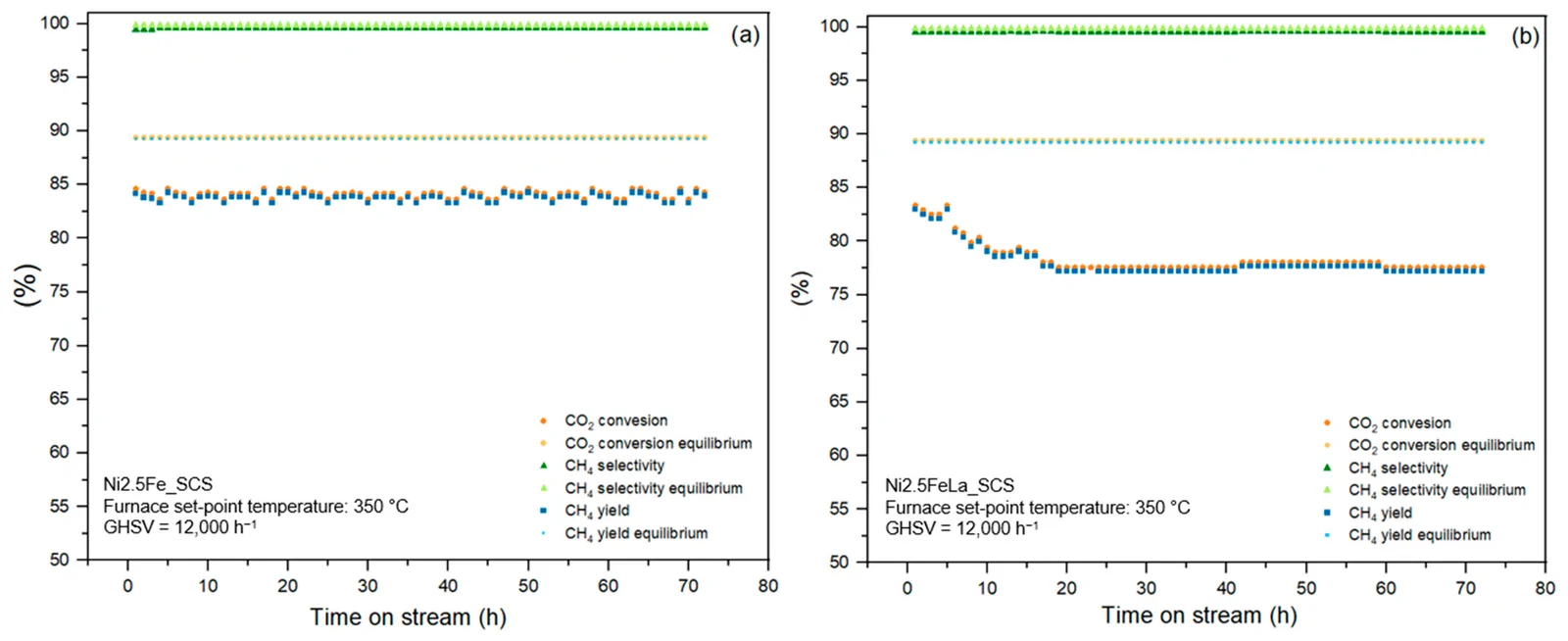
Stability test of the Ni2.5Fe_SCS (**a**) and Ni2.5FeLa_SCS (**b**) catalysts at 350 °C for 72 h (reaction conditions: 1 atm; 15 vol.% CO_2_, 60 vol.% H_2_, and 25 vol.% Ar; total flow rate = 100 mL·min^−1^; GHSV = 12,000 h^−1^; catalyst bed volume = 0.5 cm^3^; pretreatment in 5 vol% H_2_/Ar at 600 °C for 1 h).

The stability of the Ni2.5Fe_SCS and Ni2.5FeLa_SCS catalysts, as the most active samples, was tested for 72 h at 350 °C, with GHSV = 12,000 h^−1^. The catalytic performance of both samples is presented in Figure 14, while the Ni^0^ crystallite sizes after long-term stability tests are collected in Table 7. Ni2.5Fe_SCS exhibited stable performance throughout the reaction, showing no signs of deactivation. The catalyst maintained a CO_2_ conversion of approximately 83–84%, while the CH_4_ selectivity remained close to the thermodynamic equilibrium value. Consequently, the methane yield closely matched the CO_2_ conversion. On the contrary, the Ni2.5FeLa_SCS sample shows a progressive decline in activity within the first 20 h of the stability test, after which the CO_2_ conversion stabilized at 76–77% for the remaining 52 h. This decrease may be associated with changes in the accessibility or surface state of the active sites, including possible reconstruction of the Ni-Fe-La-containing surface under the reaction conditions. Importantly, the Ni^0^ crystallite size determined by XRD using the Scherrer equation remained essentially unchanged during the stability test: 4.3 nm for the reduced catalyst, 4.3 nm after 24 h on stream, and 4.1 nm after 72 h. Therefore, the absence of Ni^0^ crystallite growth suggests that the initial activity loss was not associated with thermal sintering of the Ni phase. In particular, possible accumulation of carbonate species, water-induced restructuring of the catalyst surface, or partial blocking of active sites may contribute to the initial decrease in CO_2_ conversion [81,82,83]. Tommasi et al. [81] confirmed that accumulation of strongly adsorbed carbonate/formate species may affect the availability of surface sites and CO_2_ adsorption. Mebrahtu et al. [82] studied the deactivation mechanism of hydrotalcite-derived Ni catalysts promoted with Fe at 320 °C and observed that water formed in situ in CO_2_ methanation results in the formation of nickel hydroxide (Ni(OH)_2_), leading to the formation of inactive side species. Ewald et al. [83] observed that the main deactivation mechanism for Ni-AlO_x_ mixed oxides was attributed not only to Ni particle sintering, but also a loss of BET surface area, reduction in CO_2_ adsorption capacity, and structural changes in the mixed oxide phase. Overall, these processes could affect the accessibility or surface state of the active sites without necessarily causing detectable changes in the average Ni^0^ crystallite size. However, since post-reaction characterization does not allow the exact nature of these changes to be determined, we consider this a possible explanation rather than a definitive mechanism. The subsequent stabilization of the CO_2_ conversion indicates that the catalyst reaches a relatively stable state after the initial period of operation. Notably, despite the decrease in CO_2_ conversion, the methane selectivity remained close to the thermodynamic equilibrium limit during the entire stability test; thus, the catalyst preserved its intrinsic selectivity toward methane formation. As a result, nearly all converted CO_2_ was transformed into CH_4_ during the reaction. The different stability profiles observed for the two catalysts suggest that the addition of La affected the durability of the active phase under stability tests. Additionally, the Ni^0^ crystallite size remained nearly unchanged after the stability tests (4.1–4.3 nm) compared to the reduced catalysts, indicating that no significant sintering occurred during long-term operation. This finding, particularly regarding the La-promoted methanation catalyst, is consistent with the study by Wierzbicki et al. [67] who reported that the size of Ni^0^ domains remained stable for La-containing samples after catalytic testing.

## 3. Discussion

The results obtained from complementary characterization techniques and CO_2_ methanation catalytic tests revealed distinct effects of Fe and La. In general, iron modifies the metallic active phase, which alters the electronic properties of Ni and facilitates hydrogen activation, thereby possibly enhancing the hydrogenation of CO_2_ [84]. In contrast, lanthanum enhances the surface basicity (confirmed by CO_2_-TPD) and strengthens the interaction between nickel species and the support (evidenced by H_2_-TPR). However, the simultaneous introduction of both promoters did not further improve the catalytic performance compared with the monometallic Ni_SCS catalyst, indicating that the effects of Fe and La were not simply additive.

The superior catalytic performance of the NiLa_SCS catalyst can be attributed to the combined influence of several structural and surface-related factors. The La-promoted Fe-free catalyst exhibited the smallest and well-dispersed Ni^0^ crystallites, as confirmed by XRD and EDS mapping, respectively. This was consistent with its highest H_2_-TPR reduction temperature, indicating strong metal–support interactions that limited Ni migration and coalescence during reduction. Secondly, CO_2_-TPD results indicated that La led to an increased number of medium-strength basic sites and enhanced CO_2_ adsorption. Medium-strength basic sites are considered particularly beneficial for CO_2_ methanation because they provide sufficiently strong CO_2_ adsorption, while still allowing efficient hydrogenation of the adsorbed intermediates. Moreover, based on the literature, they facilitate the formation of surface carbonate and formate species, being widely recognized as key intermediates in the hydrogenation of CO_2_ to methane [85,86]. Therefore, the superior activity of the NiLa_SCS catalyst may be attributed to the synergistic combination of highly dispersed metallic Ni, enhanced surface basicity, and strong metal–support interactions. A similar promotional effect of lanthanum was reported by Riani et al. [87], who investigated the influence of La loading on the CO_2_ methanation performance of Ni-based catalysts. Consistent with the present study, the improved catalytic activity was attributed to the enhanced basicity of the catalyst surface, stronger CO_2_ adsorption, and a reduced reaction order with respect to CO_2_. Moreover, the authors observed a nearly linear increase in catalytic activity with increasing La loading (4–37 wt.% La_2_O_3_), suggesting that further optimization of the lanthanum content may represent a promising direction for future studies on the catalytic systems developed in the present work. A similar promotional effect of lanthanum has recently been reported for Ni-La perovskite-based catalysts [88]. The authors demonstrated that strong metal–support interactions preserved under mild reduction conditions enhanced CO_2_ activation and methane formation. The studies have shown that lanthanum improves Ni dispersion, while oxygen vacancies and surface carbonate species facilitate CO_2_ adsorption. These findings are in good agreement with the present results of physicochemical characterizations.

Overall, the simultaneous incorporation of Fe and La did not provide an additional improvement in catalytic performance compared to the NiLa_SCS catalyst. In contrast, Fe alone positively affected the catalytic activity, with only a weak dependence on Fe loading, suggesting that its promotional effect was largely saturated at 2.5 wt.%. This indicates that it is primarily Fe that modifies the Ni active phase, rather than its overall loading. An additional advantage of the developed catalysts is their excellent structural stability under reaction conditions. The negligible increase in Ni^0^ crystallite size after the stability test confirms that the strong interaction between the active phase and the support effectively suppressed sintering during prolonged CO_2_ methanation. This observation is consistent with the H_2_-TPR results, which indicated strong metal–support interactions, and demonstrates that the SCS-derived catalysts possess high resistance to thermal deactivation. Based on the available literature, while Fe and La have mostly been studied independently, this study demonstrates that their combined incorporation into Ni-based catalysts does not necessarily provide a synergistic effect, despite the beneficial influence of each promoter individually.

## 4. Materials and Methods

### 4.1. Catalysts Preparation

**Figure 15:**
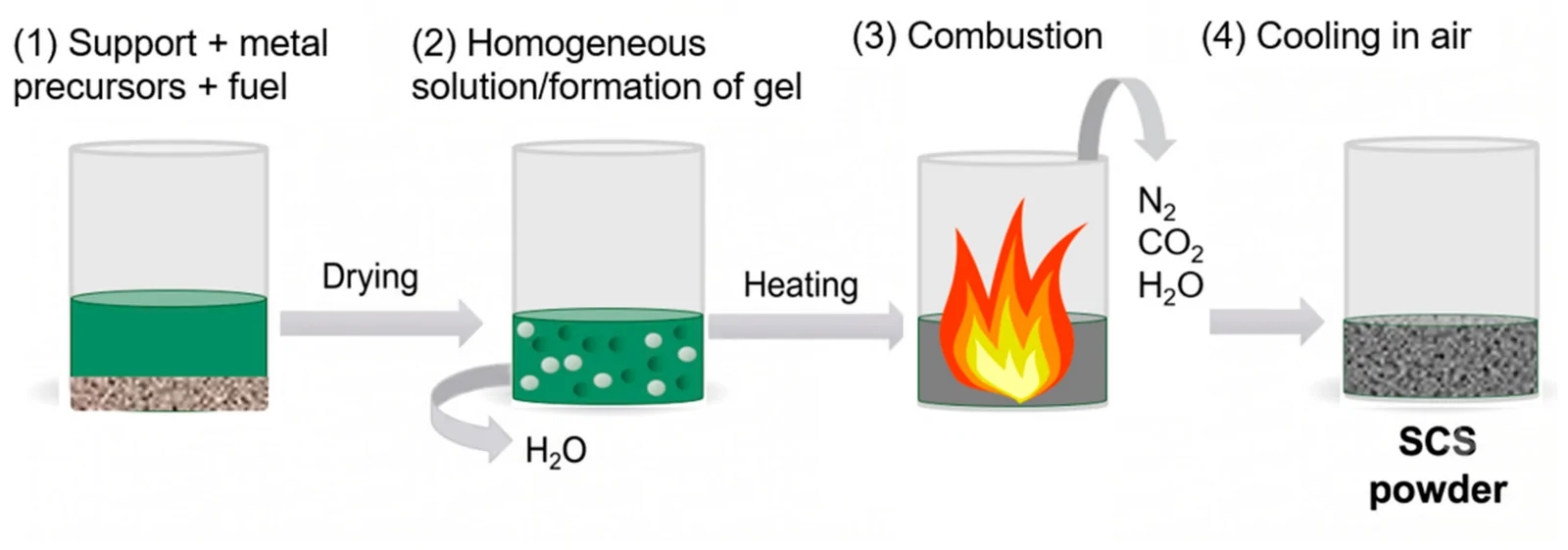
Schematic representation of the solution combustion synthesis (SCS) process.

In this research, catalysts comprising distinct precursors of metals were synthesized through a single-stage combustion process. The anticipated chemical transformations were formulated based on the oxidation-reduction valency balance of the reactive mixture. The stoichiometric amount of urea used as the fuel was calculated according to the propellant chemistry approach reported elsewhere [11,89] based on the total oxidizing and reducing valencies of the precursor mixture. In all syntheses, the fuel-to-oxidizer ratio (*φ*) was fixed at unity (*φ* = 1), corresponding to stoichiometric combustion conditions. During combustion, the metal nitrates served as oxidizers, whereas urea acted as the fuel. The highly exothermic redox reaction generated large amounts of gaseous products (CO_2_, N_2_ and H_2_O), resulting in the formation of a highly porous oxide precursor. In solution combustion synthesis (SCS), as presented in Figure 15, the fuel plays a critical role; beyond acting as the primary energy source for the reaction, it facilitates the stabilization of metal ion complexes. This stabilization enhances the solubility of the components and effectively prevents the formation of precipitates during the dehydration (water removal) phase.

Prior to catalyst synthesis, the kaolin (white kaolinite clay, Calaya) was subjected to a stepwise calcination procedure at 120 °C for 2 h, 350 °C for 3 h, and finally 600 °C for 5 h, with a heating rate of 1.5 °C/min, to induce the dehydroxylation and structural transformation of kaolinite into a predominantly disordered metakaolin phase (see XRD pattern in Figure S1). The thermal treatment conditions were selected based on the study by Liu et al. [40], which reported the highest specific surface area for the sample calcined at 600 °C. Subsequently, 25 cm^3^ of a solution containing metal nitrates (Ni(NO_3_)_2_·6H_2_O, Fe(NO_3_)_3_·9H_2_O, Mg(NO_3_)_2_·6H_2_O, Al(NO_3_)_3_·9H_2_O) and urea (all purchased from Chempur, Piekary Śląskie, Poland) was mixed with the metakaolin for 1 h at 60 °C under continuous stirring to facilitate complexation. The concentrations of the solutions were adopted to obtain 30 wt.% Ni, and 2.5, 5.0, or 7.5 wt.% Fe on kaolin, depending on the catalyst chemical composition. The mixtures were dried at 80 °C overnight, subsequently placed in ceramic boats and left for 15 min inside the furnace preheated to 350 °C. Afterwards, the samples were calcined in the following conditions: 120 °C for 2.5 h, followed by 350 °C for 3 h, 450 °C for 3 h, and 500 °C for 5 h; heating rate: 1.5 °C/min. After calcination, part of each sample was promoted with lanthanum (3 wt.%) using a dry impregnation procedure. After drying at 80 °C overnight, the La-modified catalysts were calcined at the conditions described previously. Table 8 presents the list of the prepared samples with their codes and corresponding, calculated concentration of metals.

### 4.2. Physicochemical Characterization

The physicochemical properties of the support and catalysts were characterized using the methods described below. X-ray diffraction (XRD) patterns were recorded on a Panalytical Empyrean diffractometer for as-prepared, reduced, and selected spent catalyst samples. The instrument operated in Bragg–Brentano θ-θ geometry using Cu Kα radiation (λ = 1.5406 Å). Data were collected over a 2*θ* range of 3–90°. The diffraction peaks were fitted using a Gaussian function, and the crystallite sizes were estimated from the FWHM values using the Scherrer equation. Scanning electron microscopy coupled with energy-dispersive X-ray spectroscopy (SEM/EDX) was performed using a JEOL JSM-7500F microscope (JEOL, Tokyo, Japan) equipped with an AZtecLiveLite Xplore 30 EDX system (Oxford Instruments, Abingdon, UK). Powdered samples were deposited onto carbon adhesive tape mounted on aluminum stubs. Prior to SEM–EDX analysis, all samples were coated with a 30 nm Cr layer to ensure electrical conductivity. The EDX measurements were performed at an accelerating voltage of 15 kV, an emission current of 10 µA, and a working distance of 8 mm. To ensure that the elemental composition was representative of each catalyst and not dependent on a single measurement location, EDX spectra were acquired from three different areas of each sample. These triplicate measurements were used to verify the reproducibility of the elemental composition and to assess the compositional homogeneity of the samples. Therefore, the reported composition was not based on a single-point analysis or a line scan. After confirming comparable elemental compositions across the three independently analyzed regions, EDX elemental mapping was performed on one representative region to visualize the spatial distribution of the detected elements. Nitrogen adsorption–desorption isotherms were measured at 77 K using a Micromeritics gas adsorption analyzer (Micromeritics, Norcross, GA, USA) equipped with 3Flex Surface Characterization software (version 5.03). Prior to analysis, the samples were degassed under vacuum at 30 °C for 1 h, 90 °C for 1 h, 200 °C for 6 h, and 300 °C for 3 h. The specific surface area (*S_BET_*) was determined from the adsorption branch using the BET (Brunauer–Emmett–Teller) method. Micropore volume and external surface area were estimated using the t-plot method. Mesopore size distributions were calculated from the adsorption branch using the BJH method. Temperature-programmed reduction (H_2_-TPR) was carried out using a Micromeritics AutoChem III chemisorption analyzer (Malvern Panalytical, Malvern, UK). The samples were heated from room temperature to 850 °C at a rate of 10 °C/min under a flow of 50 mL/min of 5 vol.% H_2_ in Ar. Hydrogen consumption was determined by integration of the deconvoluted H_2_-TPR profiles after baseline correction using Gaussian fitting. Quantification was performed using CuO as an external calibration standard. CO_2_ temperature-programmed desorption (CO_2_-TPD) measurements were performed using an AutoChem III instrument (software version 1.00) (Malvern Panalytical, Malvern, UK). Approximately 0.05 g of the catalyst was placed in a quartz U-tube reactor positioned inside the furnace. Helium was used as the carrier gas, 5 vol.% H_2_/Ar as the reducing gas, and 5 vol.% CO_2_/Ar as the adsorption gas. The desorbed CO_2_ was monitored during temperature-programmed desorption under a He flow. Prior to the measurements, the sample was pretreated in a He flow (30 cm^3^/min). The sample was first held at 25 °C for 80 min, then heated to 120 °C at a rate of 10 °C/min and maintained at this temperature for 60 min to remove adsorbed species. Subsequently, the sample was cooled to 50 °C under a He flow (30 cm^3^/min). The catalyst was then reduced in a flow of 5 vol.% H_2_/Ar (30 cm^3^/min at 50 °C for 60 min, followed by purging with He (30 cm^3^/min) at the same temperature for approximately 30 min. CO_2_ adsorption was carried out at 50 °C using a flow of 5 vol.% CO_2_/Ar (30 cm^3^/min). After adsorption, the sample was flushed with He (30 cm^3^/min) at 50 °C for 30 min to remove weakly physisorbed CO_2_. Finally, CO_2_ desorption was performed by heating the sample from 50 to 650 °C at a rate of 10 °C/min under a He flow (30 cm^3^/min). The XPS analyses were carried out in a PHI VersaProbeII Scanning XPS system (ULVAC-PHI, Chigasaki, Japan) using monochromatic Al Kα (1486.6 eV) X-rays focused to a 100 µm spot and scanned over the area of 400 µm × 400 µm. The photoelectron take-off angle was 45° and the pass energy in the analyzer was set to 117.50 eV for survey scans and 46.95 eV to obtain high energy resolution spectra for the C 1s, O 1s, Si 2p, Mg 1s, La 3d, Al 2s and Ni 2p regions. A dual beam charge compensation with 7 eV Ar^+^ ions and 1 eV electrons were used to maintain a constant sample surface potential regardless of the sample conductivity. All XPS spectra were charge referenced to the C-C aliphatic type bond positioned at 284.8 eV. The instrumental resolution was determined to be ~0.45 eV at 23.50 eV pass energy using the Ag 3d_5/2_ line of sputter cleaned metallic silver sample (room temperature). The operating pressure in the analytical chamber was less than 2.5 × 10^−9^ mbar. Spectra fitting was carried out using PHI MultiPak software (v.9.9.3). Spectrum background was subtracted using the Shirley method.

### 4.3. Catalytic Tests

The CO_2_ methanation catalytic tests were performed in a continuous-flow fixed-bed quartz microreactor operating under atmospheric pressure. During the catalytic tests, the furnace set-point temperature and the catalyst-bed temperature were monitored independently. The catalyst temperature was measured using a thermocouple positioned in the vicinity of the catalyst bed. Under the applied reaction conditions, the measured catalyst temperature was consistently approximately 50 °C higher than the furnace set-point temperature. Prior to the catalytic evaluation, the samples were activated under a continuous flow of 5 vol.% H_2_ in Ar (100 cm^3^/min). The reactor was heated from ambient temperature to 600 °C at a ramp rate of 10 °C/min and held at the final temperature for 1 h. This procedure ensured the complete reduction in NiO to metallic Ni^0^, while mitigating potential thermal sintering. After activation, the gas stream was immediately switched to the reaction feed, without exposing the reduced sample to air. The reacting mixture comprised CO_2_, H_2_, and Ar in a molar ratio of 1.5:6:2.5, giving the total flow rate of 100 cm^3^/min, corresponding to a gas hourly space velocity (GHSV) of 12,000 h^−1^. The catalytic performance of the samples was evaluated in the temperature range of 250–450 °C in a stepwise manner, applying a heating rate of 10 °C/min between the intervals and holding each temperature for 30 min to reach steady-state conditions. The long-term stability tests were conducted for the selected samples for 24 or 72 h at the temperature of the best catalytic performance in order to assess resistance to deactivation. The theoretical equilibrium values of CO_2_ conversion and CH_4_ selectivity were calculated using DWSIM Simulator. The conversion of CO_2_ $\left(X_{{CO}_{2}}\right)$ and selectivity to CH_4_ ($S_{{CH}_{4}}$) were calculated according to Equations (5) and (6), respectively:

(5) $$X_{{CO}_{2}}=\frac{F_{{CO}_{2},in}-F_{{CO}_{2},out}}{F_{{CO}_{2},in}}\cdot 100\%$$

(6) $$S_{{CH}_{4}}=\frac{F_{{CH}_{4}}}{F_{{CH}_{4}}+F_{CO}}\cdot 100\%$$

where *F* corresponds to the flow calculated from the concentrations of gases.

The yield of methane ($Y_{{CH}_{4}}$) resulted from the combination of CO_2_ conversion and selectivity to CH_4_ (Equation (7)):

(7) $$Y_{{CH}_{4}}=\frac{X_{{CO}_{2}}\cdot S_{{CH}_{4}}}{100\%}$$

To allow a more rigorous comparison of the catalytic performance of the investigated catalysts, the CO_2_ methanation rate was normalized to the catalyst mass and, additionally, to the BET specific surface area. The CO_2_ methanation rate normalized to the catalyst mass ($r_{{CO}_{2},m}$) was calculated according to Equation (8):

(8) $$r_{{CO}_{2},m}=\frac{{V_{in}\cdot y_{{CO}_{2},in}\cdot X}_{{CO}_{2}}}{V_{m}\cdot m_{cat}}$$

where $V_{in}$ is the inlet gas flow rate, $y_{{CO}_{2},in}$ is the molar flow rate of CO_2_ at the reactor inlet, $V_{m}$ is the molar volume of the gas (24.055 L/mol at 20 °C and 1 atm), $m_{cat}$ is the mass of the catalyst used for the reaction. The calculated rate was expressed in mmol CO_2_/(g·h). To account for differences in the specific surface area of the catalysts, the reaction rate was additionally normalized to the BET surface area ($r_{{CO}_{2},BET}$) using Equation (9):

(9) $$r_{{CO}_{2},BET}=\frac{r_{{CO}_{2},m}}{S_{BET}}$$

The BET-normalized reaction rate was expressed in mmol CO_2_/(m^2^·h). This normalization was used to assess whether differences in the observed catalytic performance could be attributed to differences in the specific surface area of the catalysts.

## 5. Conclusions

Ni-based catalysts supported on natural kaolin were successfully prepared by the solution combustion synthesis (SCS) method and promoted with Fe and/or La to investigate their performance in CO_2_ methanation. Among the investigated catalysts, the sample promoted with 3 wt.% La alone exhibited the highest catalytic activity, achieving approximately 85% CO_2_ conversion at 300 °C. This superior performance was attributed to the combination of enhanced CO_2_ adsorption, increased surface basicity, strong metal–support interactions, and the formation of small, highly dispersed Ni^0^ crystallites. The Fe-promoted catalysts also exhibited significantly improved low-temperature activity compared with the unpromoted Ni catalyst. Their enhanced performance was associated with the interactions between Ni and Fe, which modified the electronic properties of the active phase and improved its reducibility. Interestingly, the catalytic activity showed only a weak dependence on Fe loading, indicating that the beneficial effect of Fe was already achieved at the lowest investigated concentration. In contrast, the simultaneous incorporation of Fe and La did not result in additional enhancement of catalytic performance. Depending on the Fe loading, lanthanum either slightly decreased the activity or had only a negligible effect on the Fe-promoted catalysts. These findings demonstrate that the interaction between Fe and La is more complex and non-additive, despite the beneficial influence of each promoter when introduced individually.

## Figures and Tables

**Table 1:** Semi-quantitative elemental compositions of the catalysts determined by EDS.

Sample	Si (wt.%)	Al (wt.%)	Mg (wt.%)	Ni (wt.%)	Fe (wt.%)	La (wt.%)
Ni SCS	15	18	4	17	-	-
Ni 2.5 Fe SCS	15	17	3	14	2.5	-
Ni 5 Fe SCS	16	17	2	11	5	-
Ni 7.5 Fe SCS	13	16	3	12	7	-
Ni La SCS	14	17	4	18	-	4
Ni 2.5 Fe La SCS	13	16	4	17	3	3
Ni 5 Fe La SCS	14	15	3	11	4	3
Ni 7.5 Fe La SCS	13	16	3	14	7	3

**Table 2:** NiO and Ni^0^ crystallite size calculated using Scherrer’s formula.

Sample	NiO (nm)	Ni^0^ (nm)
Ni SCS	6.5	3.7
Ni 2.5 Fe SCS	6.8	4.3
Ni 5 Fe SCS	8.0	4.8
Ni 7.5 Fe SCS	6.5	5.2
Ni La SCS	3.7	3.7
Ni 2.5 Fe La SCS	6.0	4.3
Ni 5 Fe La SCS	7.6	5.9
Ni 7.5 Fe La SCS	5.2	5.3

**Table 3:** Structural and textural properties of the investigated catalysts.

Sample Code	Surface Area (m^2^/g)			Pore Volume (cm^3^/g)		Pore Size (nm)
	S_BET_^a^	S_micro_^b^	S_ext_^b^	V_micro_^b^	V_total_^c^	
Ni SCS	59	3	56	0.001	0.1	9
Ni 2.5Fe SCS	53	5	48	0.002	0.2	12
Ni 5Fe SCS	37	5	32	0.002	0.2	18
Ni 7.5Fe SCS	55	4	50	0.002	0.2	11
Ni La SCS	53	2	51	0.001	0.1	9
Ni 2.5Fe La SCS	44	9	35	0.004	0.1	13
Ni 5Fe La SCS	33	3	30	0.001	0.1	17
Ni 7.5Fe La SCS	47	1	46	~0	0.1	12

^a^ Calculated with the BET equation from the N_2_ physisorption isotherms; ^b^ Refers to the BJH single-point desorption total pore volume from the N_2_ physisorption isotherms; ^c^ Refers to the BJH desorption average pore size from the N_2_ physisorption isotherms.

**Table 4:** Deconvoluted H_2_-TPR peak temperatures and H_2_ consumption for the investigated catalysts.

Sample Code	Peak	Temperature Max. (°C)	Peak-Assigned H_2_ Consumption (mmol/g)	Total H_2_ Consumption (mmol/g)
Ni SCS	1	699	2.6	2.60
Ni2.5Fe_SCS	1	439	0.04	1.82
	2	691	1.78	
Ni 5Fe SCS	1	484	3.43	3.87
	2	686	0.46	
Ni 7.5Fe SCS	1	488	3.93	3.93
Ni La SCS	1	702	2.34	2.34
Ni 2.5Fe La SCS	1	433	0.18	1.94
	2	701	1.75	
Ni 5Fe La SCS	1	480	3.22	3.76
	2	696	0.54	
Ni 7.5Fe La SCS	1	483	3.82	3.82

**Table 5:** Surface elemental composition (in atomic %) and binding energies obtained from XPS analyses for the selected catalysts.

	Ni	La		Si	Mg	Al
Binding energy (eV)	854.7	834.3	836.1	101.8	1303.2	118.8
Assigned chemical state	Ni^2+^	La(OH)_3_	La_2_(CO_3_)_3_	silicates	Mg^2+^	Al^3+^
Ni SCS	9.9	0.0	0.0	5.2	9.6	9.8
Ni La SCS	7.3	0.5	0.3	5.6	8.2	12.2
Ni 2.5 Fe SCS	11.8	0.0	0.0	4.9	9.3	8.8

Only selected elements are presented here. Oxygen and adventitious carbon were excluded from the table for clarity.

**Table 6:** Mass- and surface-area-normalized CO_2_ conversion (350 °C, 1 atm; 15 vol.% CO_2_, 60 vol.% H_2_, and 25 vol.% Ar; total flow rate = 100 mL·min^−1^; GHSV = 12,000 h^−1^; catalyst bed volume = 0.5 cm^3^; pretreatment in 5 vol% H_2_/Ar at 600 °C for 1 h).

Sample	$r_{{CO}_{2},m}$ (mmol·g^−1^·h^−1^)	$r_{{CO}_{2},BET}$(mmol·m^−2^·h^−1^)
Ni_SCS	87.17	1.48
Ni2.5Fe_SCS	102.55	1.93
Ni5Fe_SCS	93.79	2.53
Ni7.5Fe_SCS	89.77	1.63
NiLa_SCS	135.27	2.55
Ni2.5FeLa_SCS	135.12	3.07
Ni5FeLa_SCS	117.56	3.56
Ni7.5FeLa_SCS	122.66	2.61

**Table 7:** Ni^0^ crystallite size before and after the stability test calculated using Scherrer’s formula.

Sample	Ni^0^ (nm)
Ni 2.5 Fe SCS reduced	4.3
Ni 2.5 Fe SCS spent (24 h)	4.3
Ni 2.5 Fe SCS spent (72 h)	4.1
Ni 2.5 Fe La SCS reduced	4.3
Ni 2.5 Fe La SCS spent (24 h)	4.3
Ni 2.5 Fe La SCS spent (72 h)	4.1

**Table 8:** List of prepared samples.

Sample Code	Sample Description
Ni_SCS	Kaolin modified with 30 wt.% Ni
Ni2.5Fe_SCS	Kaolin modified with 30 wt.% Ni and 2.5 wt.% Fe
Ni5Fe_SCS	Kaolin modified with 30 wt.% Ni and 5 wt.% Fe
Ni7.5Fe_SCS	Kaolin modified with 30 wt.% Ni and 7.5 wt.% Fe
NiLa_SCS	Kaolin modified with 30 wt.% Ni, impregnated with 3 wt.% La
Ni2.5FeLa_SCS	Kaolin modified with 30 wt.% Ni and 2.5 wt.% Fe, impregnated with 3 wt.% La
Ni5FeLa_SCS	Kaolin modified with 30 wt.% Ni and 5 wt.% Fe, impregnated with 3 wt.% La
Ni7.5FeLa_SCS	Kaolin modified with 30 wt.% Ni and 7.5 wt.% Fe, impregnated with 3 wt.% La

## Data Availability

The data presented in this study are available from the corresponding author upon reasonable request.

## Supplementary Materials

The following supporting information can be downloaded at: https://www.mdpi.com/article/10.3390/molecules31183218/s1, Figure S1. XRD patterns of kaolin before (KNC) and after calcination (MK), with the identified crystalline phases present in the clay. Figure S2. XRD patterns obtained for the calcined samples: kaolin modified with Ni and Fe (a), kaolin modified with Ni, Fe, and La (b). Figure S3. XPS spectra for the reduced Ni_SCS. NiLa_SCS. and Ni2.5Fe_SCS: Mg 1s region (a), Si 2p region (b), Al 2s region (c). Figure S4. Survey X-ray photoelectron spectra of the investigated catalysts: Ni_SCS (a), NiLa_SCS (b), and Ni2.5Fe_SCS (c).

## Abbreviations

The following abbreviations are used in this manuscript:

BE	Binding Energy
CO_2_-TPD	Carbon Dioxide Temperature-Programmed Desorption
DRM	Dry Reforming of Methane
EDS	Energy-Dispersive X-ray Spectroscopy
H_2_-TPR	Hydrogen Temperature-Programmed Reduction
ICDD	International Centre for Diffraction Data
IUPAC	International Union of Pure and Applied Chemistry
RWGS	Reverse Water-Gas Shift
S_BET_	Specific Surface Area Determined by the Brunauer–Emmett–Teller Method
SCS	Solution Combustion Synthesis
SEM	Scanning Electron Microscopy
S_EXT_	External Surface Area
S_micro_	Micropore Surface Area
TCD	Thermal Conductivity Detector
V_micro_	Micropore Volume
V_total_	Total Pore Volume
WGS	Water-Gas Shift
XPS	X-ray Photoelectron Spectroscopy
XRD	X-ray Diffraction
