# Supplementary material for: Enhanced CO2 Methanation over Solution-Combustion Synthesized Ni/Kaolin Catalysts: The Effect of Fe and La Promotion
Source: Molecules. 2026 Sep 11;31(18):3218. doi: 10.3390/molecules31183218 (PMC13608972; doi:10.3390/molecules31183218)
Supplement: Supplementary file 1 [file molecules-31-03218-s001.zip › molecules-4505009-supplementary materials.pdf]

SUPPLMENTARY MATERIALS

Enhanced CO_2 Methanation over Solution-Combustion Synthesized Ni/Kaolin Catalysts: The Effect of Fe and La Promotion

Agnieszka Szymaszek-Wawryca [1,*], Szymon Hanf [1], Michał Szymaszek [1], Konrad Świerczek [1], Dorota Duraczyńska [2], Mateusz Marzec [3] and Monika Motak [1]

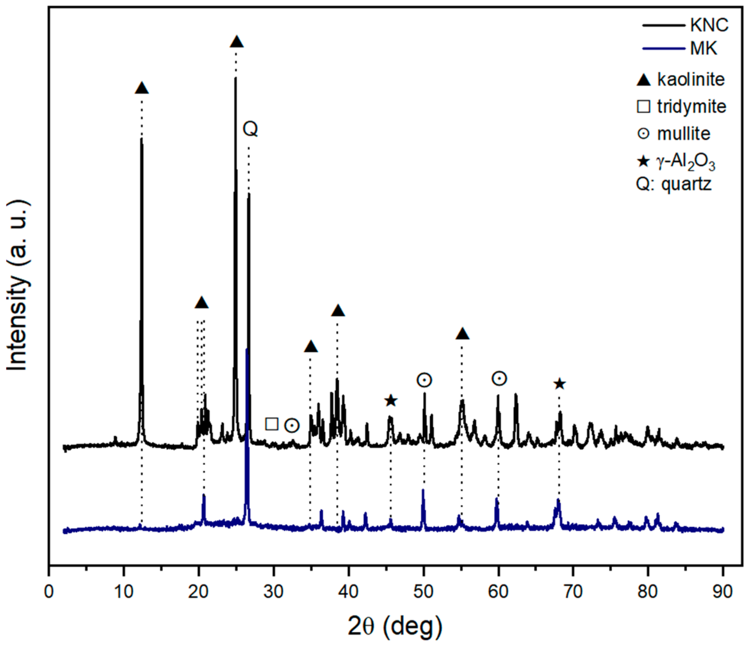

Figure S1. XRD patterns of kaolin before (KNC) and after calcination (MK), with the identified crystalline phases present in the clay.

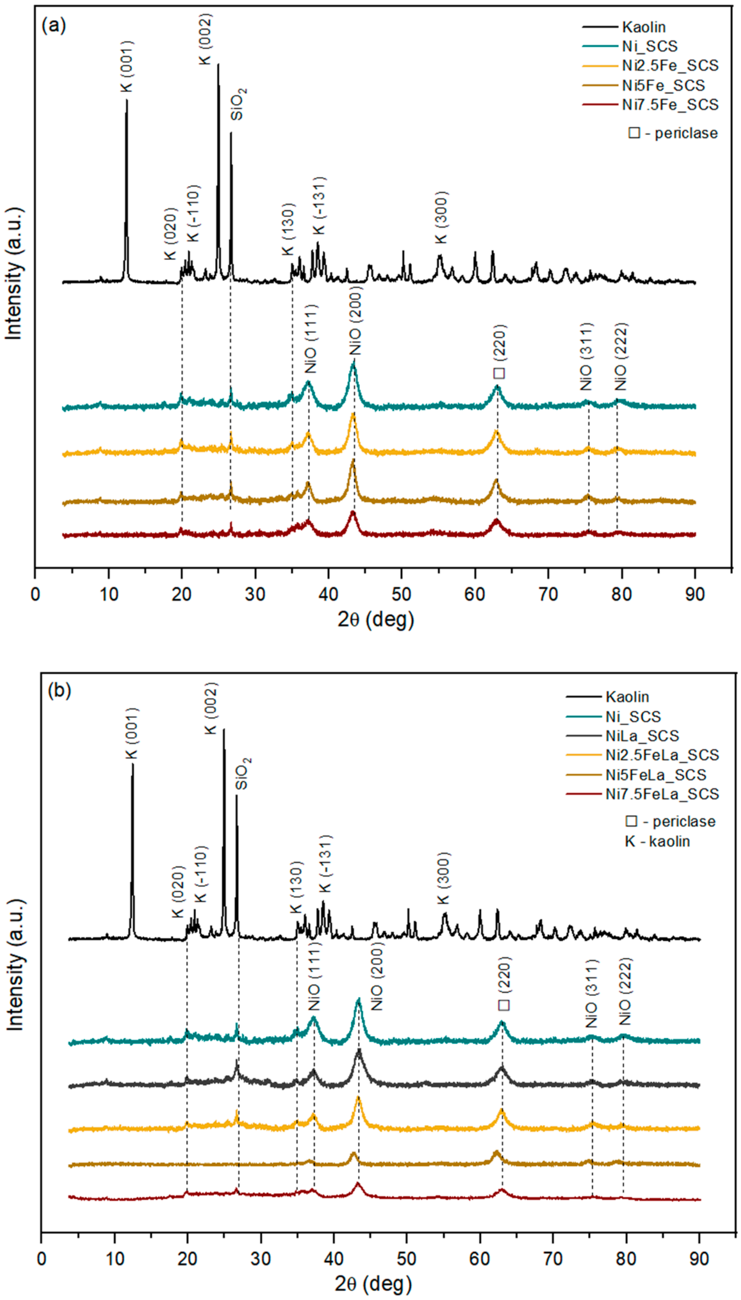

Figure S2. XRD patterns obtained for the calcined samples: kaolin modified with Ni and Fe (a), kaolin modified with Ni, Fe, and La (b).

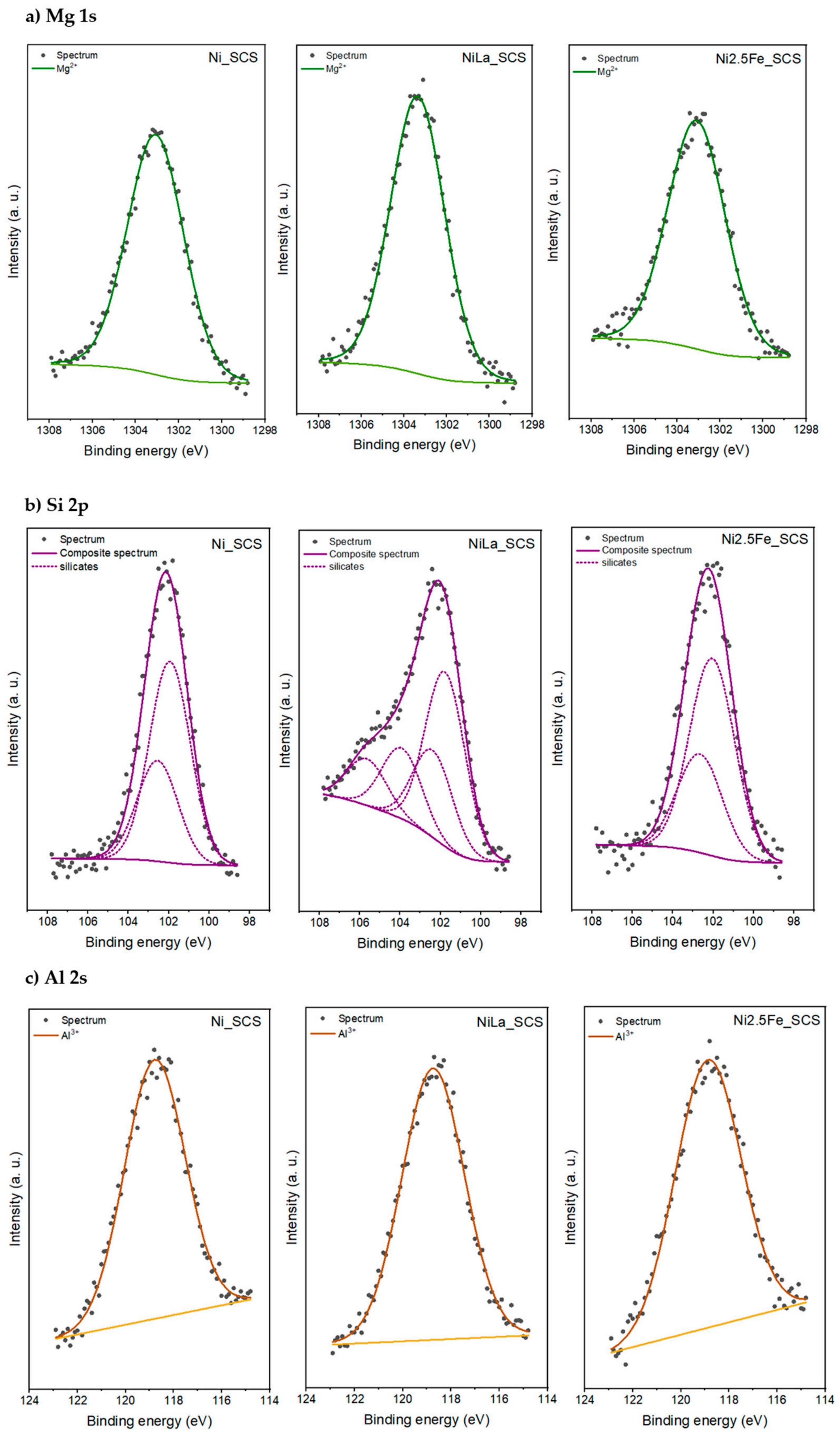

Figure S3. XPS spectra for the reduced Ni_SCS, NiLa_SCS, and Ni2.5Fe_SCS: Mg 1s region (a), Si 2p region (b), Al 2s region (c).

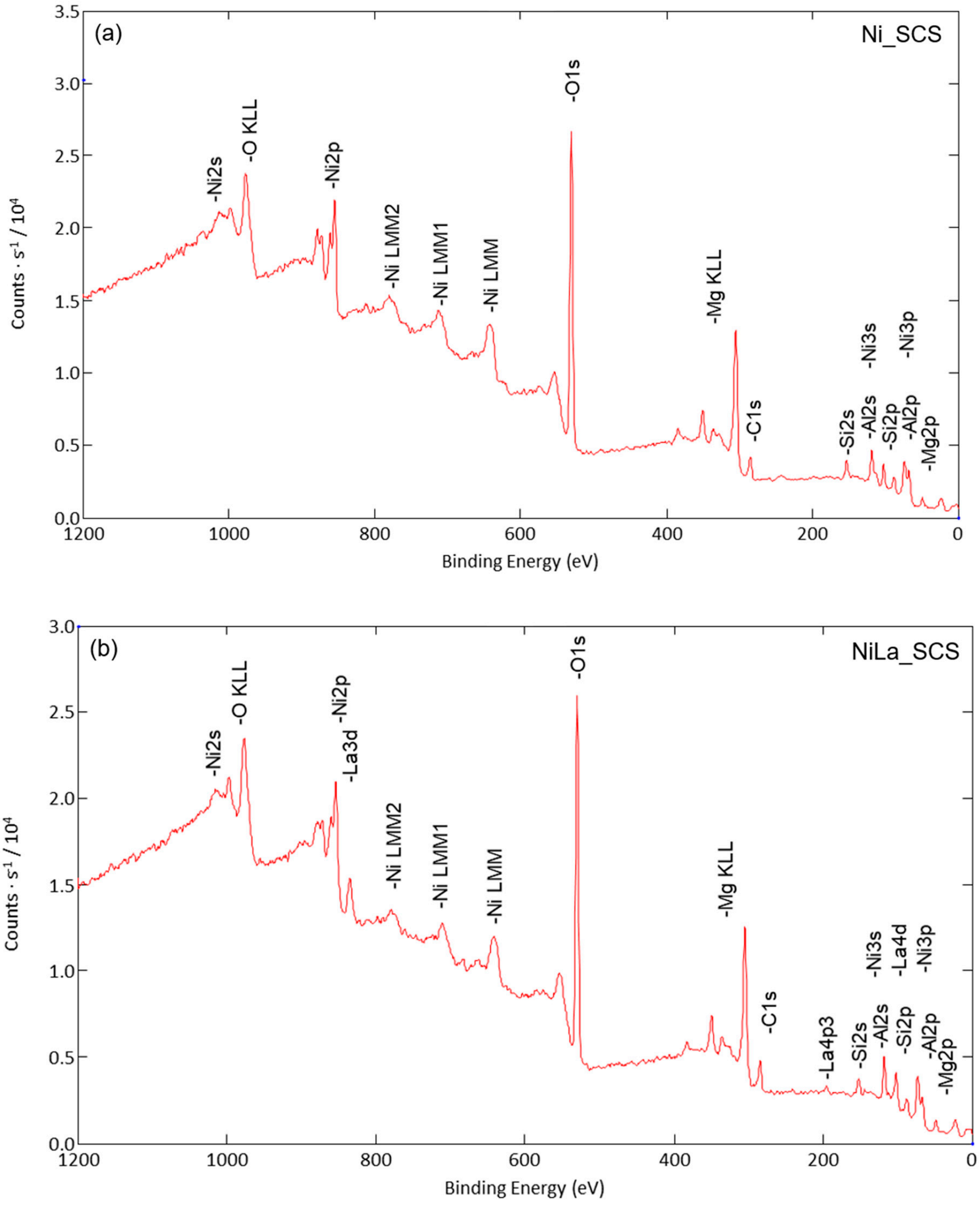

Figure S4. Survey X-ray photoelectron spectra of the investigated catalysts: Ni_SCS (a), NiLa_SCS (b), and Ni2.5Fe_SCS (c).

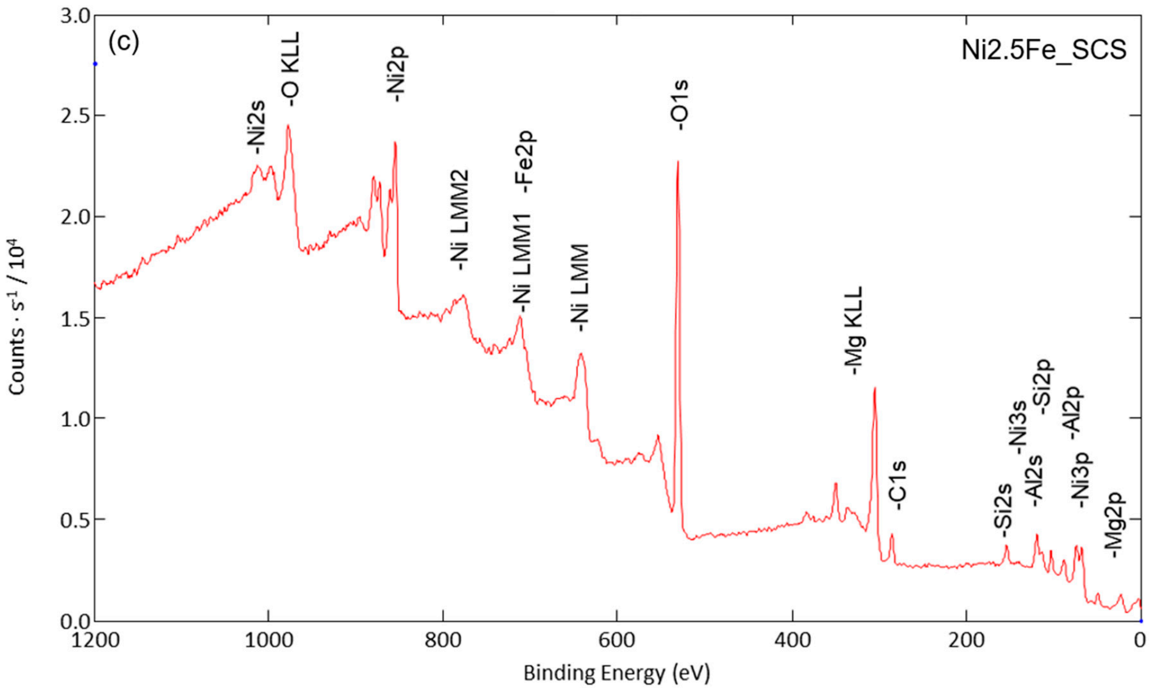

Figure S4. *cont*. Survey X-ray photoelectron spectra of the investigated catalysts: Ni_SCS (a), NiLa_SCS (b), and Ni2.5Fe_SCS (c).
